# Assessing the drivers of gut microbiome composition in wild redfronted lemurs via longitudinal metacommunity analysis

**DOI:** 10.1038/s41598-022-25733-x

**Published:** 2022-12-12

**Authors:** Tatiana Murillo, Dominik Schneider, Michael Heistermann, Rolf Daniel, Claudia Fichtel

**Affiliations:** 1 Behavioral Ecology and Sociobiology Unit, German Primate Center, Göttingen, Germany; 2 Genomic and Applied Microbiology and Göttingen Genomics Laboratory, Institute of Microbiology and Genetics, Georg-August-University of Göttingen, Göttingen, Germany; 3 Research Center for Tropical Diseases (CIET) and Faculty of Microbiology, University of Costa Rica, San José, Costa Rica; 4 Endocrinology Laboratory, German Primate Center, Göttingen, Germany

**Keywords:** Microbial ecology, Animal behaviour, Microbial communities, Metagenomics, Microbiome

## Abstract

The gut microbiome influences host’s immunity, development, and metabolism and participates in the gut–brain axis, thus impacting the health of the host. It is a dynamic community varying between individuals and within individuals at different time points. Hence, determining the factors causing this variability may elucidate their impact on host’s health. However, understanding the drivers of variation has proven difficult particularly as multiple interactions occur simultaneously in the gut microbiome. We investigated the factors shaping the gut microbiome by applying the metacommunity concept where the gut microbiome is considered as a microbial community shaped by the interactions within the community, with the host and microbial communities outside the host, this through a longitudinal study in a wild primate. Focal behavioral data were collected for 1 year in four groups of redfronted lemurs to determine individual social and feeding behaviors. In addition, regular fecal samples were collected to assess bacteria, protozoa, and helminths through marker gene analysis and to measure fecal glucocorticoid metabolite (fGCM) concentrations to investigate the impact of physiological stress on the gut microbiome. Higher consumption of leaves and elevated fGCM concentrations correlated with higher alpha diversity, which also differed among groups. The major drivers of variation in beta diversity were group membership, precipitation and fGCM concentrations. We found positive and negative associations between bacterial genera and almost all studied factors. Correlations between bacterial indicator networks and social networks indicate transmission of bacteria between interacting individuals. We detected that processes occurring inside the gut environment are shaping the gut microbiome. Host associated factors such as, HPA axis, dietary changes, and fluctuations in water availability had a greater impact than interactions within the microbial community. The interplay with microbial communities outside the host also shape the gut microbiome through the exchange of bacteria through social relationships between individuals and the acquisition of microorganisms from environmental water sources.

## Introduction

The gut microbiome are the prokaryotic and eukaryotic communities inhabiting the host’s gastrointestinal tract playing a pivotal role in the health of the host^1–3^. This community is dynamic, varying between individuals, and within an individual at different time points^4,5^. Hence, identifying the drivers of gut microbiome variability will help to understand how its fluctuations may associate with health outcomes^5,6^. However, detecting these drivers has proven difficult as few studies recognize the gut microbiome as an ecological system^7^. Furthermore, longitudinal studies capturing the dynamics of the gut microbiome are rare or based on only few individuals resulting in limited data^5^. The metacommunity concept recognizes the gut microbiome as an ecological system in which multiple interactions occur simultaneously, thereby providing a framework for determining its drivers^6,7^. The metacommunity concept states that the local community assemblage is shaped by several processes, including factors shaping the niche, in this case the gut system, and the interaction with other microbial communities outside the host through dispersal of microorganisms^7–9^. Here, we investigated the drivers of the gut microbiome in a wild primate longitudinally by applying metacommunity concepts by addressing the impact of the interactions with microbial communities outside the host and environmental selection in the gut niche. Thus, considering it as a changing ecosystem shaped by factors inside and outside the host simultaneously.

In gut microbiome research, dispersal processes of the microorganisms between hosts and the environment can be assessed through social interactions and habitat sharing^5,10^. Group membership in wild non-human primates and cohabitation in humans are predictors of gut microbiome similarity^11–16^. Furthermore, the host’s social behaviors can also predict gut microbiome similarity^17–20^. Environmental selection for gut communities occurs in the intestinal niche through feedbacks between the host and the microorganisms and amongst microorganisms^6,7^. Host-associated factors such as, age, sex, and physiological stress, i.e., hypothalamic–pituitary–adrenal (HPA) axis activation, may influence immunity and intestinal physiology altering the gut microbiome^1,2,21^. Furthermore, shifts in the host’s diet impact gut bacterial communities as they alter nutrient availability^22–25^. Gut inhabitants interact between themselves through trophic chains, predation, and competition for resources^3,26^. For instance, in non-human primates, higher bacterial alpha diversity correlates to higher eukaryotic diversity^27^. Therefore, the presence of helminths and/or protozoa may impact the abundances of bacterial taxa^28–30^. Despite being challenging, research on wild animals provide an exceptional possibility to apply metacommunity concepts for investigating the drivers of the gut microbiome in undisturbed scenarios^5,8^.

We examined the drivers of the gut microbiome by applying metacommunity concepts in a longitudinal setup in wild redfronted lemurs in Kirindy Forest, Madagascar. These lemurs live in small multifemale-multimale groups consisting of individuals of different ages allowing to estimate the potential impact of sex and age^31,32^. Kirindy Forest is a highly seasonal environment with a cold dry season with almost no precipitation (April–October) and a short warm rainy season (November–March)^33^. These seasonal changes affect food availability, meaning redfronted lemurs must shift their diets^25,34^. Moreover, fluctuations in precipitation reduce the availability of drinking water^35,36^. HPA axis activation due to exposure to stressors has been previously investigated in these redfronted lemurs through standardized measurement of fecal glucocorticoid metabolites (fGCM)^37–39^. For instance, during the dry season and in periods of social instability such as the mating (May–June) and the birth (September–October) season individuals have higher fGCM concentrations indicating the activation of their HPA axis^38,40,41^. Furthermore, these lemurs harbor diverse protozoa and helminths in their guts, which can be assessed through marker gene analysis to investigate microbe-microbe interactions^25,42,43^. Finally, behavioral observations of wild primates provide the opportunity to estimate the effects of direct and indirect social contacts in dispersal processes of microbes within a group^10^. Particularly, in redfronted lemurs that perform auto- and allogrooming with a buccal structure, i.e., the toothcomb^44^. Oral grooming may increase the possibility of up taking microorganisms from their own fur and the fur from other individuals in comparison to manual grooming which is exhibited in anthropoid primates^37^. Altogether, these lemurs provide a unique possibility to study some of the multiple drivers of the gut microbiome in a wildlife setting.

We investigated (a) the interactions between the host and the microorganisms, (b) the interplay between gut prokaryotes and eukaryotes and (c) dispersal processes of bacteria within and between groups and the environment as drivers of the gut microbiome in a longitudinal study using a dense sampling regime. Focal behavioral data and monthly fecal samples (N = 799) were collected during 1 year from all individuals (N = 35) belonging to four groups. Bacteria, protozoa, and helminths were identified with marker gene analysis and fGCM measurements were performed to determine HPA axis activation. Furthermore, precipitation was measured as a proxy for changes in available water sources. We hypothesized that (1) host intrinsic factors such as sex, age, and fGCM concentrations as well as extrinsic factors such as precipitation, and diet impact gut microbiome composition and diversity. We anticipate that increased levels of fGCM, lower age and higher precipitation correlate with lower alpha diversity, while higher consumption of leaves correlates with higher alpha diversity^22,23,25,45^. No impact of sex is expected to be detected^15^. (2) Protist and helminth richness are predicted to correlate with changes in bacterial diversity and composition, but no associations with particular bacteria are predicted as these are parasite species specific^3,26,28,29^. (3) The gut microbiome composition and beta diversity, but not alpha diversity, are foreseen to differ among groups and a higher diversity of social interactions is predicted to correlate positively with alpha diversity^12–16^. (4) Bacterial indicator networks of amplicon sequence variants (ASVs) correlate with social networks indicating bacterial transmission through social interactions.

## Methods

### Sample, behavioral, and environmental data collection

This study was conducted at the research station of the German Primate Center in Kirindy Forest, Western Madagascar (44° 39′ E, 20° 03′ S) from May 2018 to April 2019^33^. Samples and data were collected over 1 year from 35 redfronted lemurs belonging to four groups consisting of varying number of individuals (A: 5–8 individuals, B: 5–10 individuals, F: 6–7 individuals, and J: 11 individuals). During the study period four individuals left group B (BAdoF, BRinF, BBurM, BBorF), one left group F (FGozM), one individual migrated from group B to A (BTilM), and one individual emigrated from and immigrated into the same group over a period of nine months (AAmoM) (Supplementary Table S1). 799 fecal samples (1 to 5 samples per individual per month; Supplementary Fig. S1) were collected in RNAlater (Thermofisher Scientific, Massachusetts, USA) from the forest floor immediately after defecation between 7:30 and 11:00, stored at − 20 °C in the field station and later at − 80 °C in Germany (Supplementary Table S1). 641 of these samples were splitted and 0.5–1.0 g of feces were placed in 5 mL of 80% ethanol for measuring fGCM concentrations using validated methodologies (see below). Behavioral data was collected by continuous focal observations for 30 min in the morning (7:30–11:00) and afternoon (14:00–17:00). Feeding behaviors were recorded by protocolling the duration and the ingested food item (leaves, flowers, or fruits). For social interactions, we protocolled the duration of grooming and body contact, and the interacting partners. Fecal samples and behavioral data were collected in a randomized but counter-balanced order. Precipitation was collected with a Tropos data logger (Lambrecht meteo, Göttingen, Germany) and we calculated the mean precipitation 30 days prior to sample collection according to previous publications^22^.

### Behavioral data analysis

A total of 1042 h of behavioral data was collected. For each fecal sample we estimated the following behaviors during the 30 days prior to collection^22^: (a) the proportion of time the individual spent feeding either on fruits, flowers or leaves, from the total observation time on those 30 days and (b) a social interaction diversity index: $(Shannon \; diversity \; of \; social \; interactions*Average \;number\; of \; interactions\; per \;dyad)$ for each individual, accounting for the number of interacting partners and duration of these interactions. This index increases with the average dyadic interaction time and when the interactions are more evenly distributed among dyads.

### DNA extraction, amplification and sequencing of taxonomic marker genes

DNA extractions were performed from 150 mg fecal sample following the manufacturer’s instructions but including a bead beating step of 6.5 m/s and 24 × 2 for 20 s using FastPrep-24 5G (MP Biomedicals, California, USA) with the PowerSoil DNA isolation kit (Qiagen, Hilden, Germany). For amplification of the 16S rRNA gene (Supplementary Table S1), each sample was amplified separately, whereas for the 18S rRNA gene monthly samples were pooled together before amplification (Supplementary Table S2). PCR reactions for both taxonomical marker genes were performed in triplicates with the primers and thermocycling protocols listed in the Supplementary Table S3 and included a negative control without DNA template and a positive control^46,47^. Triplicates per sample were pooled equimolarly and purified with the MagSi-NGS PREP Plus magnetic beads according to the manufacturer instructions (Steinbrenner Laborsysteme GmbH, Wiesenbach, Germany) in the Janus Automated Workstation (Perkin Elmer, Waltham Massachusetts, USA). Ilumina MiSeq sequencing adapters were attached using the Nextera XT Index kit (Ilumina, San Diego, USA). Index PCRs were performed with 5µL of template, 2.5 µL of each index primers, 12.5 µL of 2 × KAPA HiFi HotStart Ready Mix and  2.5 µL PCR grade water with a thermocycling scheme of 95 °C for 3 min, 8 cycles of 30 s at 95 °C, 30 s at 55 °C, 30 s at 72 °C and a final elongation at 72 °C for 5 min. Indexed products were purified as previously and quantified with the Quant-iT dsDNA HS assay kit in a Qubit fluorometer (Invitrogen GmbH, Karlsruhe, Germany). Sequencing was performed in the Göttingen Genomics Laboratory in the MiSeq Ilumina platform with a read length of 2 × 300 bp using dual indexing and reagent kit v3 (600 cycles) as recommended by the manufacturer. To control for batch effects random samples were resequenced in different runs and only differences to the range of technical replicates were detected.

### Bioinformatic processing of amplicon data

Paired-end reads were quality-filtered with fastp v0.20.0 using default settings plus an increased per base phred score of 20, base pair corrections by overlap (-c), as well as 5′- and 3′-end read-trimming with a sliding window of 4, a mean quality of 20 and minimum sequence length of 50 bp. Quality-controlled reads were merged with PEAR v0.9.11 and primer-clipping was performed with Cutadapt v2.5 with default settings. VSEARCH 2.14.1 was used for size-sorting, size-filtering (16S rRNA ≥ 300 bp; 18S rRNA ≥ 250 bp) and dereplication. The sequences were denoised with UNOISE3 using default settings and chimeras were removed with UCHIME3 (de novo followed by reference-based) leading to the final set of amplicon sequence variants (ASVs). 16S rRNA were mapped against the ASVs and taxonomy was assigned with a minimum identity of 70% using BLAST 2.9.0 + against the SILVA SSU 138.1 NR^48^. Best hits were only accepted if coverage ≥ 90 and blastn hit identities were corrected to unclassified according to the thresholds proposed by^49^. 18S RNA reads were assigned using BLAST 2.9.0+ against the PR2 database^50^ and taxonomy was determined with the Bayesian LCA-based Taxonomic Classification Method (BLCA) using a confidence score threshold of 0.80^51^. To control for spurious reads and index hopping, ASVs with < 0.25% reads were removed before analysis^52^. All sequencing statistics are in Supplementary Table S4.

### Measurement of fecal glucocorticoid metabolites

Glucocorticoid metabolites (fGCMs) were extracted from the fecal samples directly at the field site using a validated method^53^ applied successfully in previous lemur studies^54,55^. Briefly, for fGCM extraction, tubes with the collected feces were vortexed for 2 min to homogenize the fecal matter within the 80% ethanol and the fecal suspension was centrifuged with a manually operated centrifuge (Hettich GmbH & Co. KG Tuttlingen, Germany) for 10 min. 1.5 mL of the extracts were collected into 2 mL polypropylene tubes and stored in the field at ambient temperature in the dark and at − 20 °C in Germany. To calculate the weight of each fecal sample collected, a differential weighting of the samples prior to and after the collection of the feces was undertaken. FGCM concentrations were determined using an enzyme immunoassay (EIA) for the analysis of immunoreactive 11-oxoetiocholanolone, a group-specific measurement of cortisol metabolites in primates^39^. The EIA, carried out as described in^38^, has been validated for tracking HPA axis activity in redfronted lemurs^37,38^. Inter- and intra-assay coefficients of variations (CVs) of high- and low-value quality controls were 10.9% (high, n = 52) and 9.7% (low, n = 52) and 6.8% (high, n = 17) and 8.2% (low, n = 17), respectively. FGCM values are expressed as mass per gram of wet fecal weight (ng/g).

### Data analysis and statistics

Data visualization and statistical analysis were performed using R v4.1.0 and RStudio v1.4.1717 with ampvis2, ape, stringr, reshape2, viridis, data.table, tidyverse, and ggplot2. All data for alpha and beta diversity analysis of 16S rRNA data was rarefied to the lowest read counts whereas for barcharts, linecharts, and network estimation it was normalized using GMPR (Supplementary Table S4). Bacterial alpha diversity was calculated as Faith´s phylogenetic diversity (PD) with picante using a phylogenetic tree generated by aligning all sequences with MAFFT v7.407-1 at 100 iterations, calculated using FastTreeMP v2.1.7 and midpoint-rooted using FigTree v1.4.4.

#### Analysis of gut protozoa and helminth

Helminthic and protozoan gut communities were studied by amplifying the V4 region from the 18S rRNA gene. ASVs from previously reported gut protozoa and helminth were extracted from the 18S rRNA gene data to remove environmental contaminants. The analyzed taxa were *Trichostomatia*, *Nematoda*, *Metamonada*, *Coccidiomorphea*, and *Cestoda*^27,42,56^. Samples were merged per individual per month and parasite richness was estimated as the number of observed ASVs. A Jaccard matrix was calculated to investigate changes in parasite beta diversity and visualized with a Principal Coordinate Analysis (PCoA) in ampvis2. A PERMANOVA test to estimate beta diversity variation due to group, sex, age, and season was calculated with the adonis function from the vegan package using individual as strata to account for repeated sampling, 10,000 permutations and Benjamini–Hochberg FDR correction.

#### Testing the factors affecting bacterial alpha diversity

The effects of group, sex, age, social interactions, parasite richness, feeding on fruits, flower or leaves, and precipitation on PD were tested by fitting a Linear Mixed Model (LMM) with lme4. To achieve normally distributed and homogenous residuals PD was Box-Cox transformed (as a log transformation was not sufficient in this case). Test predictors were group, sex, age, social interactions, and parasite richness, whereas diet, and precipitation were control predictors. Age was log-transformed to achieve a more symmetrical distribution and to avoid influential cases, and all predictors were z-transformed to facilitate model convergence without affecting the shape of the distribution. Individual identity was included as random intercept effect and the random slopes for all fixed effects (except for group and sex) into individual identity were included to keep the type I error at the nominal level of 5%^57^. Correlations between random intercepts and random slopes were included. The significance of the test predictors was determined by calculating a null model excluding all test predictors and comparing it to the full model using a likelihood ratio test. The effects of single fixed effects were determined with the package lmerTest. Homogeneous and assumptions of normally distribution of residuals were checked visually with QQ-plots of residuals and plotted against fitted values revealing no obvious deviations. Calculation of Variance Inflation Factors using car was done on a model lacking all random effects and no issues of collinearity were detected (maximum:1.433). Model stability was determined by dropping predictors one at a time, fitting a full model from each of the subsets and comparing the estimates of these models to those obtained for the initial full model revealing it was acceptable. The same model was calculated for those samples having fGCM measurements by adding log-transformed fGCM values as a test predictor to avoid influential cases, as it was skewed otherwise. No collinearity was detected (maximum:1.404) and model stability was also acceptable.

#### Drivers of bacterial beta diversity dissimilarities

Weighted UniFrac matrices (WUnifrac) were calculated in ampvis2 and visualized with PCoA. To estimate the drivers of beta diversity variation, PERMANOVA tests were calculated with the adonis function from the vegan package using individual as strata to account for repeated sampling, by runnning 10,000 permutations and performing Benjamini–Hochberg FDR corrections. Three different datasets were tested: (a) diet and social interactions (n = 773), (b) parasite richness (n = 682) and c) fGCM levels (n = 547) as for some samples either behavioral or parasite data was missing and PERMANOVA cannot be calculated in samples with missing data points. Group, sex, age, and precipitation were tested in all datasets.

#### Associations between bacterial genera and all covariates

Associations of group, sex, age, social interactions, diet, precipitation and fGCM concentrations to bacterial genera were determined using the package MaAsLin2^58^. Two models with the random effect of individual were calculated: (a) all factors without fGCM levels (n = 799) and (b) all factors including fGCM concentrations (n = 641). ASV counts were centered-log ratio transformed and Benjamini–Hochberg FDR corrected p-values were reported.

#### Bacterial indicator and social network analysis

Bacterial indicator networks were calculated with indicspecies to identify correlations between ASVs abundances and individuals^59^. multipatt was used to determine the phi coefficient of association and the association strength between an ASV and an individual using 999 permutations. Networks were visualized in Cytoscape v3.8.2 using the individuals and their associated bacterial taxa as nodes, whereas edges represent those significant (*p* < 0.05) correlation coefficients between nodes. The networks had an edge-weighted spring embedded layout, taxon node size was adjusted according to taxa abundance, edge width represents association strength to target, and all nodes and edges were bundled. Undirected weighted social networks for each group were calculated using the Dyadic Sociality Index (DSI)^60^ including proportion of grooming, and body contact behaviors during the whole study, and visualized with igraph. Previously, correlations between both behaviors were determined with Mantel correlations tests. For group F and J, no correlations were detected, but for uniformity the DSI was also used. Correlations of the number of shared indicative ASV and the DSI between individuals were estimated with Mantel tests.

#### Bacterial community comparison between longtime residents and migrating individuals

To investigate the differences on bacterial community composition of migrating individuals of groups A, B and F, those samples from months when all individuals were present were extracted and merged for each individual per month. This was done to calculate a PCoA analysis from a WUnifrac distance matrix (as done previously) and the first coordinate of the PCoA against sampling month was plotted.

### Ethics statement

We performed non-invasive collection of fecal samples. All methods were performed in accordance with the relevant guidelines and regulations, including the ARRIVE guidelines. This research was approved and authorized by the Ministry of the Environment from Madagascar, the Mention Zoologie et Biodiversité Animale Université d'Antananarivo and the CNFEREF Morondava (N°245/17/MEEF/SG/DGF/DSAP/SCP.re, N° 47/18/MEEF/SG/DGF/DSAP/SCP.re, and N° 215/18/MEEF/SG/DGF/DSAP/SCP.re).

## Results

### Bacterial, protozoan, and helminthic communities of redfronted lemurs

**Figure 1:**
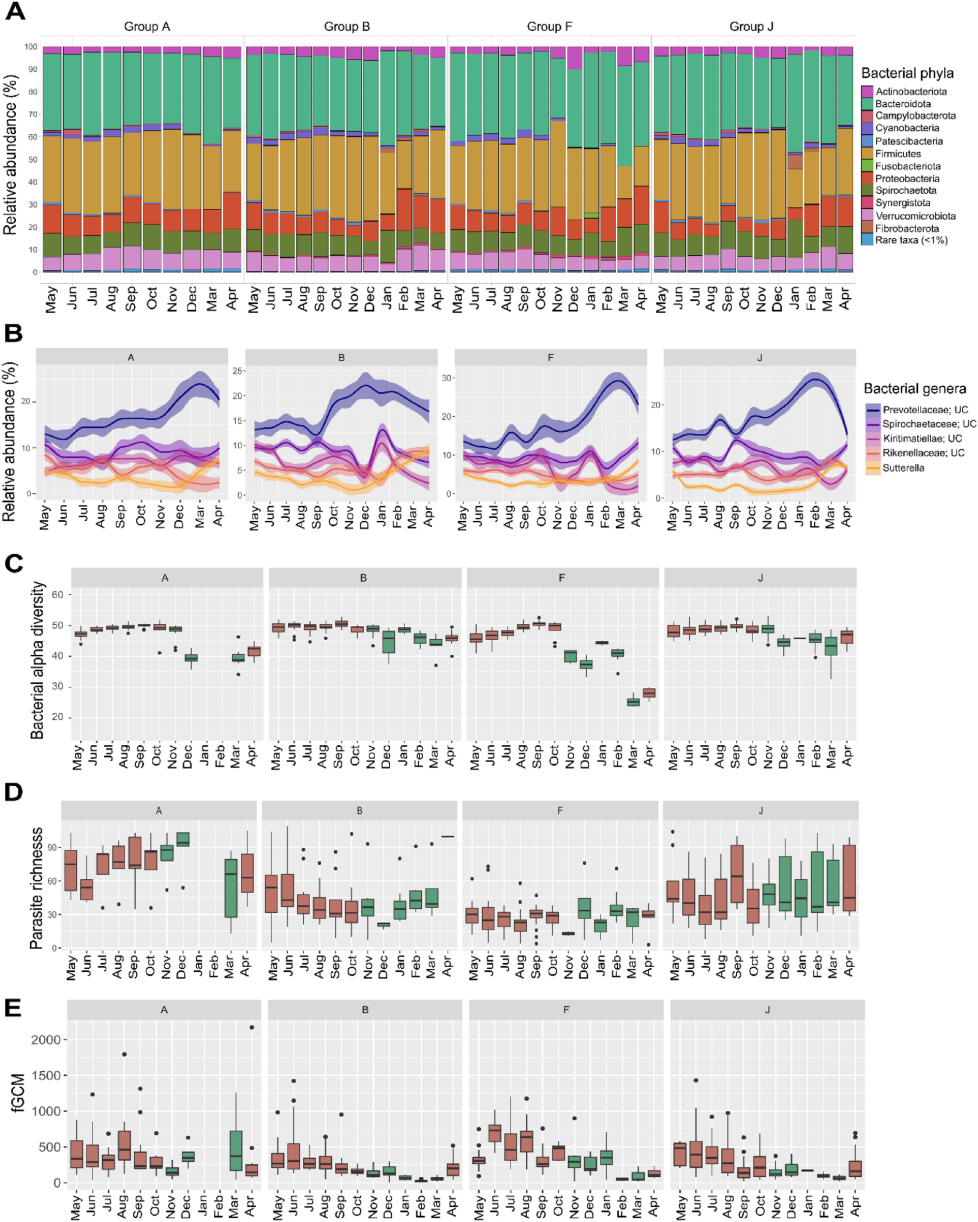
Overview of the monthly fluctuations of bacterial communities, bacterial alpha diversity, eukaryote parasite richness and fGCM concentrations for each lemur group. Box plots are color coded to indicate the dry (brown) and rainy (green) season. (**A**) Monthly averaged relative abundances of bacterial phyla per group. (**B**) Top 5 most abundant bacterial genera and their monthly changes. (**C**) Monthly variations in alpha diversity measured as Faith’s Phylogenetic Diversity Index. (**D**) Monthly changes in parasite richness. (**E**) Concentrations of fGCM measured as ng/g of wet feces aggregated per month.

The five most abundant bacterial phyla showed consistent relative abundances in all four groups: Bacteroidota (35.49% ± 3.24), Firmicutes (30.01% ± 4.60), Proteobacteria (9.83% ± 3.00), Spirochaetota (9.41% ± 1.43) and Verrucomicrobiota (7.02% ± 1.01) (Fig. 1A, Supplementary Table S5). On genus level the five most abundant bacteria were also consistent among all groups with variations in their abundances during the sampling period (Fig. 1B). Although four genera could not be classified at genus level, they belong to the families *Prevotellaceae* (16.26% ± 5.75), *Spirochaetaceae* (9.33% ± 3.20), *Rikenellaceae* (6.62% ± 3.53) and *Kiritimatiellae* (5.44% ± 2.66) while the fifth most abundant genus was *Sutterella* (3.64% ± 2.62). Bacterial alpha diversity calculated as Faith’s Phylogenetic diversity index (PD) had similar monthly trends in all groups (Fig. 1C). Lower PD was detected in April for all groups towards the transition between rainy and dry season (A: 42.14 ± 5.67; B: 43.31 ± 4.44; F: 30.19 ± 7.33; J: 40.92 ± 8.24) whereas higher PD was observed in October in the transition from dry to rainy season (A: 50.40 ± 0.91; B: 50.49 ± 0.93; F: 48.96 ± 0.72; J: 50.18 ± 1.63).

Regarding the helminthic and protozoan gut communities, all amplified taxa were *Metazoa* including *Nematoda* (48.40% ± 10.69), *Craniata* (5.53% ± 4.22) and *Arthropoda* (2.98% ± 2.63), *Streptophyta: Embryophyceae* (21.86% ± 6.25), *Fungi: Ascomycota* (1.44% ± 1.07) and *Basidiomycota* (3.85% ± 6.59), *Ciliophora: Litostomatea* (9.50% ± 6.44) and *Metamonada: Trichomonadea* (1.24% ± 0.72) with a total of 4.04% ± 2.40 unclassified reads (Supplemental Fig. S2A, Supplementary Table S6). Further on, only eukaryote orders formerly reported as inhabitants of the gut of humans or animals were analyzed. The orders detected were *Chromadorea*; *Nematoda* (A: 79.38% ± 17.04; B: 78.02% ± 22.00; F: 73.55% ± 28.44; J: 79.22% ± 21.37), *Trichostomatia*; *Litostomatea* (A: 19.49% ± 15.92; B: 15.95% ± 17.63; F: 16.82% ± 21.87; J: 18.59% ± 20.68), and *Trichomonadida*; *Trichomonadea* (A: 1.13% ± 2.80; B: 6.02% ± 16.28; F: 9.61% ± 21.33; J: 2.17% ± 4.43) present in all individuals (Supplemental Fig. S2B). Except for *Litostomatea*, which was not detected in one individual from August until October. Subsequently, we determined the number of observed ASVs for the same taxa as a measure of parasite richness. Parasite richness showed variations between groups, individuals, and months (mean ± SD number of ASVs: group A: 71.44 ± 24.95; group B: 45.43 ± 26.27; group F: 27.44 ± 13.14; group J: 49.51 ± 26.44) (Fig. 1D). A PERMANOVA based on a Jaccard matrix showed that the factor explaining the highest variance on parasite richness was season (r^2^ = 0.011, *p* = 0.001) (Supplemental Fig. S2C and Supplementary Table S7). Parasite richness differed between groups and season.

The highest concentrations of fGCM were detected in August for group A (571.9 ng/g ± 412.65), and in June in all other groups (B: 447.00 ng/g ± 373.13; F: 706.33 ng/g ± 177.87; J: 463.23 ng/g ± 337.29) (Fig. 1E). Consumption of leaves, fruits and flowers varied across months and between groups (Supplementary Fig. S3A). December and January were the months with the highest precipitation (Supplementary Fig. S3B).

### Factors driving changes of bacterial alpha diversity

**Figure 2:**
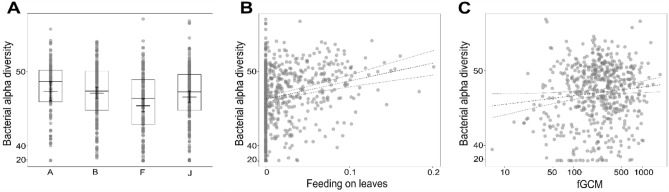
Effects of group membership, consumption of leaves and concentrations of fGCM on bacterial alpha diversity measured as PD. (**A**) Group membership. (**B**) Proportion of time feeding on leaves during the 30 days prior to sampling. (**C**) Log-transformed fGCM concentrations given in ng/g feces.

We analyzed the effects of sex, age, group membership, social interactions, parasite richness, dietary changes, and precipitation on alpha diversity measured as PD. Although the full-null model comparison was significant only by trend (LMM: Estimate = 26.124, SE = 1.641, *t*-value = 15.915, model comparison: *p* = 0.088, Supplementary Table S8) an effect of group membership, with group F having a lower alpha diversity compared to the other groups (*p* = 0.009, Fig. 2A) was detected. Additionally, feeding on leaves correlated positively with alpha diversity (*p* = 0.000, Fig. 2B). The second model for alpha diversity had a reduced dataset (see “Methods”) including fGCM concentrations. Similarly, an effect of group membership for group F and feeding on leaves was detected (LMM: Estimate = 26.786, SE = 1.506, t-value = 17.782, full-null model comparison: *p* = 0.038; Supplementary Table S9). FGCM concentrations correlated positively with alpha diversity (*p* = 0.027, Fig. 2C) with higher fGCM concentration resulting in a higher alpha diversity. No effects of sex, age, social interaction diversity index, or parasite richness were detected.

### Factors leading to dissimilarities between gut bacterial communities

**Figure 3:**
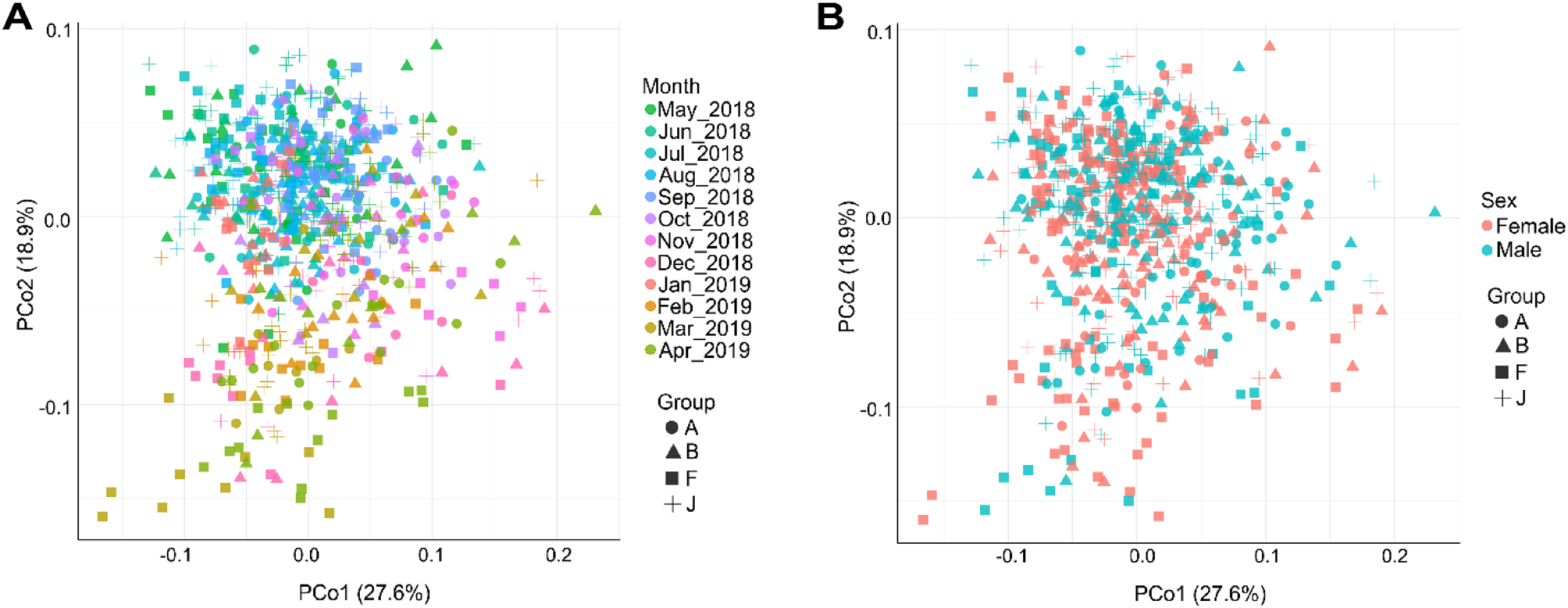
PCoA from Weighted Unifrac matrices of the bacterial community denoting beta diversity changes. (**A**) Data points color coded for the different study months to depict monthly changes in beta diversity. (**B**) Data points color coded for sex. Groups are depicted in A and B by symbols.

To estimate the drivers of variance on beta diversity, PERMANOVA based on WUnifrac matrices on three different datasets were calculated due to missing data points (see “Methods”). The factors tested in the first dataset explained 8.9% of the variance (Fig. 3A,B), with group (r^2^ = 0.035, *p* < 0.000) and precipitation (r^2^ = 0.021, *p* < 0.000) being the strongest predictors (Supplementary Table S10). In the second dataset (Supplementary Table S11) including the parasite data, the total variance explained was 10.4% with group (r^2^ = 0.041, *p* < 0.000) and precipitation (r^2^ = 0.024, *p* < 0.000) as strongest predictors. Finally, in the dataset including fGCM concentrations (Supplementary Table S12) 14.5% of the variance was explained with fGCM (r^2^ = 0.028, *p* < 0.000), group (r^2^ = 0.052, *p* < 0.000) and precipitation (r^2^ = 0.022, *p* < 0.000) as strongest predictors.

### Associations of social interactions, parasite richness, fGCM concentrations, diet, and precipitation to bacterial genera composition

**Figure 4:**
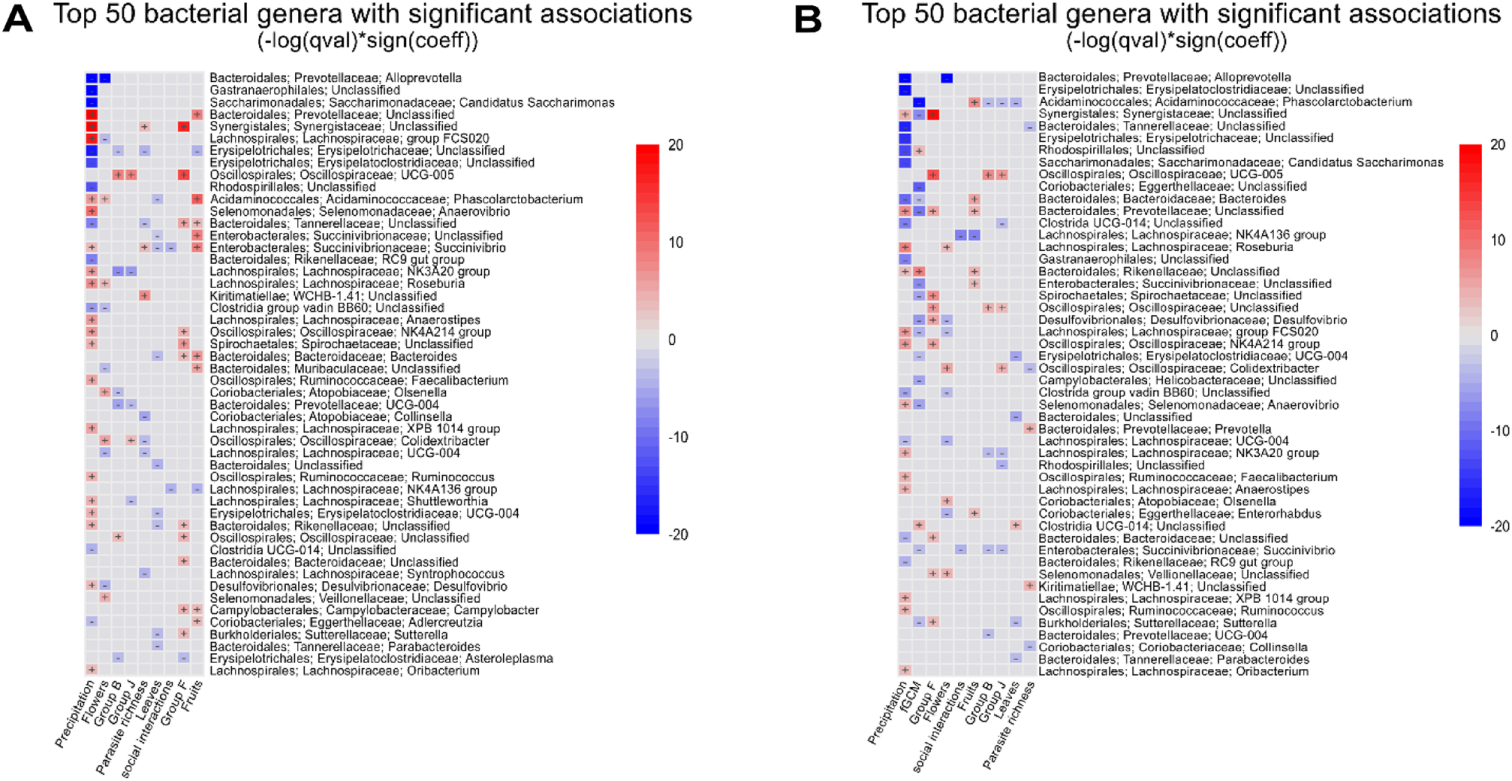
Top 50 most abundant bacterial genera associated with group, social interactions, age, sex, parasite richness, diet, and precipitation. Association directions are color coded positive (red) and negative (blue). (**A**) Full dataset. (**B**) Reduced dataset including fGCM concentrations. Group A was the reference category for group comparisons.

A total of 50 bacterial genera were associated with group, social interaction diversity index, feeding on flowers, leaves or fruits, parasite richness, age, and precipitation in the full dataset (Fig. 4A and Supplementary Table S13). Precipitation and diet had the most associated taxa, with 33 and 36 genera, respectively. Dispersal processes attributed to group membership and social interactions had 27 and 2 associated taxa, respectively. Parasite richness was associated with 12. In the subsetted dataset including fGCM concentrations, 50 genera associated with at least one of the studied covariates (Fig. 4B and Supplementary Table S14). Twenty taxa associated with fGCM levels, whereas slight variations were detected for the other covariates: precipitation^26^, diet^24^, group^28^, social interactions^2^, and parasite richness^5^. In both datasets, no genus associations with sex and age were detected.

### Correlation between social networks and bacteria indicator networks

**Figure 5:**
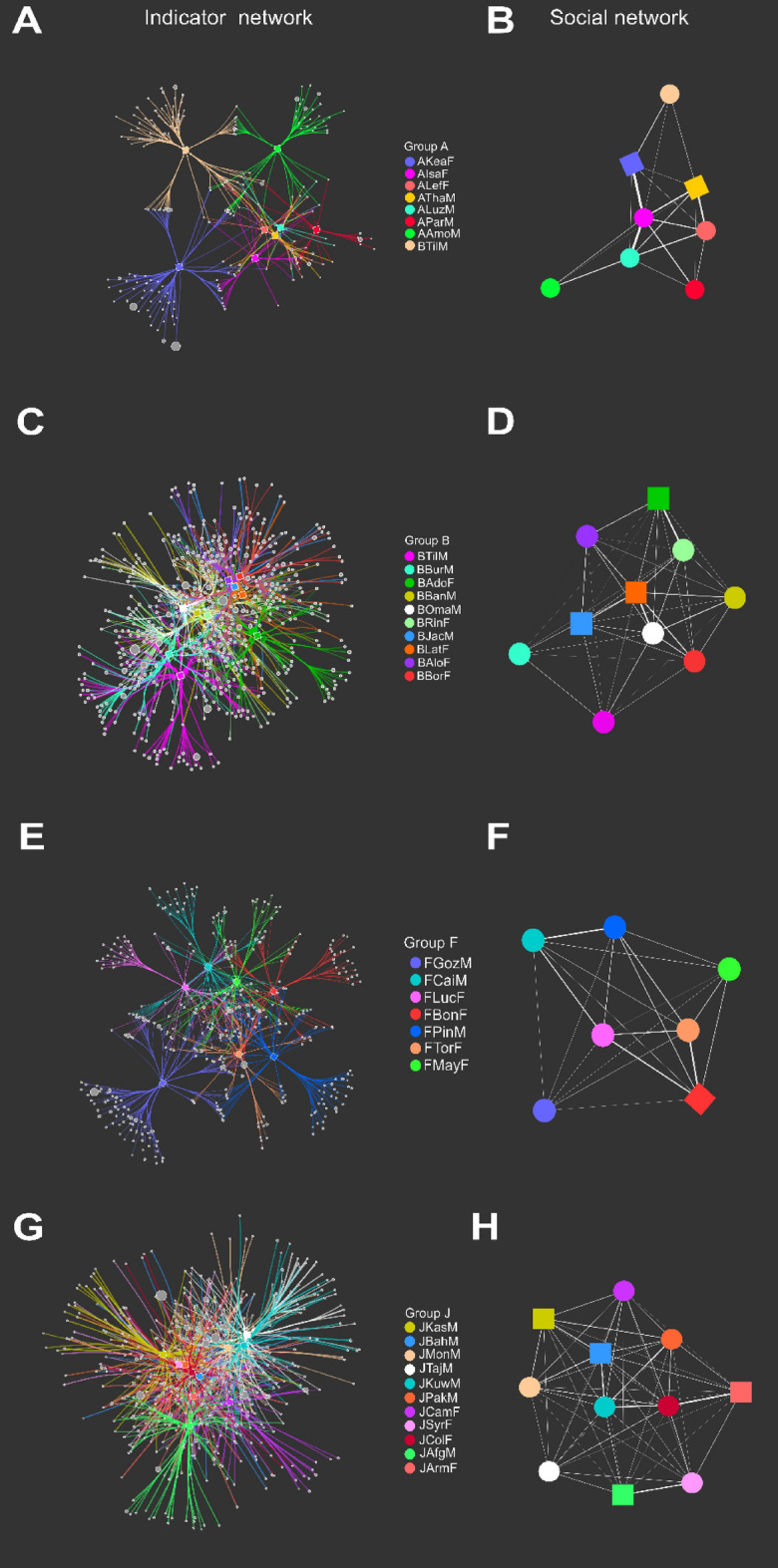
Indicative networks and social networks for the individuals of each group based on ASVs. Networks were colored by individual; nodes are shaped in the social network according to adult (circle) or juvenile/infant (square). Bacterial indicator ASV network and social network of group A (**A** and **B**), group B (**C** and **D**), group F (**E** and **F**) and group J (**G** and **H**).

To determine if sharing of bacterial ASVs between individuals correlates to an individual’s social network, bacterial indicator networks were calculated. These networks were determined based on ASVs to identify bacterial ASVs whose relative abundance significantly correlate within and between individuals and, hence, indicate microbe dispersal through social interactions. Correlations between bacterial indicator ASVs and social networks were detected for group A (r^2^ = 0.536, *p* = 0.002, Supplementary Tables S15 and S16), and B (r^2^ = 0.399, *p* = 0.013, Supplementary Tables S17 and S18), but not for group F (r^2^ = 0.502, *p* = 0.089, Supplementary Tables S19 and S20) and J (r^2^ = 0.235, *p* = 0.060, Supplementary Tables S21 and S22) (Fig. 5G,H). Furthermore, individuals who emigrated from groups: A (AAmoM; Fig. 5A,B), B (BTilM; Fig. 5C,D), and F (FGozM; Fig. 5E,F) had less strong social relationships and a more differentiated bacterial indicator network profile than individuals that remained in the groups. One individual, BTilM, immigrated to group A, thus showing fewer connections in the social network, and shared less ASVs with other group members. Monthly fluctuations in bacterial community composition of the migrating individuals compared to longtime residents of each group were further explored in Supplementary Fig. S4. In group A, the two immigrating individuals, BTilM and AmoM, were more distant from others (Supplementary Fig. S4A). In addition, the bacterial communities of BAdoF and BTilM were more different in the last sampling month of residency in group B (Supplementary Fig. S4B). Finally, the bacterial community of the emigrating male FGoZM was clearly different from the rest of the group (Supplementary Fig. S4C).

## Discussion

Our longitudinal study revealed that host-microbe interactions, the interplay between bacteria and parasite richness, and dispersal processes of bacteria through social relationships impact the fluctuations of the gut microbiome. From the investigated host-associated factors, the HPA axis measured through fGCM concentrations revealed the strongest impact. Higher fGCM levels correlated with higher alpha diversity and associated with changes in bacterial abundances. Conversely, no impact of age and sex was identified. Interactions between eukaryotes and bacteria were detected. Parasite richness explained only a small amount of variance in beta diversity but impacted both, positively and negatively, the abundances of specific bacterial genera. Dispersal processes of bacteria between hosts were estimated from social interactions and group membership. Group membership explained 3–5% of the variance in beta diversity, groups had different alpha diversity, and each group had its own associated bacterial genera. Diversity of social interactions explained only low variance in beta diversity but impacted the abundances of certain bacteria. In two of the four groups, an individual’s social network correlated to sharing of significantly associated bacterial ASVs with other individuals, suggesting transmission of taxa through social interactions.

### The HPA axis is an important driver of gut microbiome variation in wild lemurs

Higher fGCM concentrations, indicating HPA axis activation, correlated to increased bacterial alpha diversity. The highest mean fGCM values for three of the four groups were detected during June indicating an influence of the mating season on HPA axis activity^40,61^. However, for one group the highest fGCM values were recorded for August, a period when redfronted lemurs are exposed to environmental stressors due to reduced food and water availability^33,34,38^. Even though environmental stressors could have increased fGCM levels, we suspect that social stressors had a greater influence, as reported before in these lemurs^38^. Our longitudinal approach aiming to capture these periods when redfronted lemurs experience social and environmental challenges made it possible to detect this impact^33,34,38^. Studies in other species, in contrast, revealed no correlations or negative correlations between glucocorticoids and alpha diversity^62–65^. Increased fGCM levels may, however, result in higher bacterial alpha diversity due to the down regulation of the immune response controlling the gut microbiome by glucocorticoids, thus allowing the colonization by other taxa^66,67^. Consumption of leaves during the dry season also correlated with higher alpha diversity which may contribute to a certain degree to the positive correlation between fGCM concentrations and alpha diversity. However, redfronted lemurs fed more on leaves in September/October, whereas fGCM concentrations peaked in June/August, indicating that feeding on leaves and fGCMs influence separately alpha diversity. In addition, fGCM concentrations was one of the covariates explaining most variation in beta diversity, indicating that fGCM concentrations drive differences in beta diversity. Positive associations were detected only with three genera from *Rikenellaceae*, *Rhodospirillales* and *Clostridia*. Higher abundances in genera from *Clostridia* have been reported in mice exposed to social stressors and western lowland gorillas with high fGCM measurements^62,65^. Fourteen genera were impacted negatively by fGCM concentrations, including genera from *Prevotellaceae*, *Spirochaetaceae* and *Sutterella*, some of the most abundant taxa detected in redfronted lemurs^25^. Genera from *Prevotellaceae* are important for the digestion of plant polysaccharides^68^, while treponemes from non-human primates harbor genes for the digestion of sucrose and glycerophospholipids^69^.This suggests that HPA axis activation can impact bacteria providing relevant pathways for food digestion. Furthermore, a negative influence on the most abundant bacterial genera may also lead to an increase in alpha diversity due to changes on the gut community allowing other genera to thrive. A negative association to a genus from *Helicobacteraceae*, a potential pathogen, was also detected in yellow-legged gull^45^. The activation of the HPA axis and its production of glucocorticoids can influence the gut microbiome through the increase of gut permeability allowing the translocation of bacteria from the lumen to other tissues^63^. Also, HPA axis activation can reduce immune activation and increase susceptibility to infections by pathogens^63,70,71^. Our results indicate that social stressors from the mating season like reproductive competition and female evictions can activate the HPA axis impacting the gut microbiome^40,72^.

### Diversity of gut protozoa and helminths impact the bacterial community

Helminths and protozoa were prevalent all year in almost all individuals, and the orders detected coincide with our previous study of redfronted lemurs^25^. Variations in eukaryotic communities between samples were explained by season. Our results support previous reports from redfronted lemurs that detected seasonal differences in the abundances of *Chromadorea*, and protozoa diversity^73^. Parasite richness only explained very low variation in bacterial beta diversity but associated positively and also negatively with certain bacterial taxa, supporting other studies from non-human primates^28–30^. Positive associations with *Succinivibrio* and Verrucomicrobiota have been reported in humans as well^74,75^. Helminthic intestinal infections can increase mucus production, thus influencing positively mucin utilizers, such as Verrucomicrobiota^23,76^. Also, negative associations of genera from *Lachnospiraceae* such as *Syntrophococcus* and XPB-1014 group, have been detected in humans with helminthic and helminthic-protozoan infections^74,75,77^. *Lachnospiraceae* are fiber metabolizers essential for the digestion of the lemur’s diet, particularly during increased leaf consumption^78,79^. Other negatively associated taxa like *Collinsella*, *Colidextribacter*, *Tannerellaceae* and *Erysipelotrichaceae* are gut bacteria with no association to parasites reported so far^80–82^. Each parasite can have specific effects on the gut niche, thus explaining that parasite richness explains only a low amount of beta diversity since all parasites were investigated together^3,76,83^. Also, it was not possible to compare infected vs. uninfected individuals, as parasites were prevalent in almost all individuals all year. We investigated only presence and absence of parasites, as abundance estimations from 18S rRNA should be taken cautiously^43^. Parasites can impact bacteria positively or negatively through trophic chains, predation, competition, and immunomodulation^3,26,76^. These are all processes that could be occurring in redfronted lemurs due to their diverse eukaryotic communities, thus providing a unique study system for future investigations of transkingdom interactions.

### Dispersal processes between hosts are drivers of gut microbiome community composition

Group membership, diversity of social interactions and social networks were used to estimate bacterial dispersal through social behaviors. Group membership was one of the covariates explaining the highest variance in beta diversity and having the most associated taxa, indicating that each group has a specific bacterial community despite fluctuations of the gut microbiome. Group differences in the gut microbiome can be due to sharing of microorganisms through social interactions between group members, as it has been proposed previously^12–16,84^. Differences in bacterial communities can also be explained by habitat use, but all studied groups have overlapping home ranges with at least one group^85^. However, group F, occupies a home range more distant to a river traversing the study area, which may affect the habitat quality and could explain the differences in alpha diversity^33,35,86^. Kinship may also influence group differences but not all group members were related thus, we suppose that it may have a lower impact^17,40^. Diversity of social interactions only explained very low variance in beta diversity, but it had negatively associated taxa. *Succinivibrio*, a starch degrader, was impacted negatively indicating that social interactions can impact genera carrying out relevant metabolic functions^87^. Correlations between bacterial indicator ASVs and social networks indicate that at least some of these indicator taxa are shared through affiliative interactions. Hence, individuals exhibiting strong social relationships, share bacterial ASVs through their affiliative behaviors influencing bacterial presence and abundances. The fact that no correlations were detected for groups F and J indicate that this signal is harder to detect in groups with less differentiated social relationships. Less ASVs were shared by group members that emigrated or immigrated the groups possibly due to their short residency in the group as reported in baboons^84,88^. Correlations between social networks and gut microbiome similarity have been reported in other wild primates^17,19^, but this study is the first to analyze the impact of social networks on bacterial ASV level.

### Ecological determinants of variations in gut bacterial communities

Feeding on flowers, fruits, or leaves, and precipitation correlated to changes in beta diversity and had positive and negative associated taxa with each of them. Consistent with a previous study in these lemurs and other research, feeding on leaves correlated with a higher alpha diversity^22,23,25^. Changes in precipitation had the most associated taxa. Precipitation affects the availability of water sources in the habitat of redfronted lemurs between dry and rainy season^25,33,36^. During the rainy season redfronted lemurs drink water from temporal puddles, tree holes or the river, whereas during the dry season only water ponds in the river remain^35,89^. We speculate that changes in water intake due to reduced water availability may impact the gut microbiome by influencing gut transit times, affecting clearance of microorganisms during excretion, and determining the availability of nutrients and water in the gut habitat^35,90^. Some studies suggest that in humans stool consistency is the strongest predictor of gut microbiome composition and it is relevant as it indicates differences in water availability and activity in the colon influencing the gut niche^90,91^. However, it is also possible that the lemurs ingested bacteria from water sources, and this uptake results in fluctuations in the gut microbiome according to the water sources available^22,89^. The type of food item consumed is another important driver of bacterial community composition as they are also their main energy source^92,93^. The capacity of the gut microbiome to adapt to dietary changes is essential for the acquisition of nutrients from food by the host^8^. This effect was detected when shifting from a diet based on leaves, which is composed of complex polysaccharides, to a diet based on flowers and/or fruits, which is rich in mono- and disaccharides, coinciding with our previous study^25,94^. This impact was detected despite not quantifying precisely the amount of food items consumed, a common limitation of fieldwork studies.

## Conclusion

The gut microbiome of wild redfronted lemurs is shaped by group membership, social interactions, fGCM levels, diet, precipitation, and parasite richness at different intensities. Thus, bacterial dispersal processes between hosts and the environment, plus selection by the gut niche through prokaryotic-eukaryotic interactions, changes in water availability, diet fluctuations, and the host’s HPA axis activation impact the gut microbiome. Furthermore, we detected an influence of HPA axis activation and parasite richness on bacteria genera important for digestion and energy harvest from diet. Our results demonstrate the importance of longitudinal studies with dense sampling regimes to capture the fluctuations of the gut microbiome as an ecosystem. This approach enabled us to detect the periods when each of the factors impacted the gut microbiome asserting that both processes outside and inside the host influence simultaneously its dynamics.

## Supplementary Information

Supplementary Information 1.

Supplementary Table S1.

Supplementary Table S2.

Supplementary Table S5.

Supplementary Table S6.

Supplementary Table S13.

Supplementary Table S14.

Supplementary Table S15.

Supplementary Table S17.

Supplementary Table S19.

Supplementary Table S21.

## Data Availability

Raw reads were deposited in the NCBI Sequence Read Archive under the Bioproject PRJNA694983 (https://www.ncbi.nlm.nih.gov/bioproject/?term=PRJNA694983) (Supplementary Table S1 and Supplementary Table S2). The datasets generated and analyzed during the current study are available in figshare: https://figshare.com/projects/Multiscale_study_of_temporal_drivers_of_gut_microbiome_composition_in_wild_redfronted_lemurs/126316. All R scripts can be found in https://github.com/tmurillocorrales/Redfrontedlemurs_gutmicrobiome.

## Abbreviations

ASVs: Amplicon sequence variants
fCGM: Fecal glucocorticoid metabolites
PCoA: Principal coordinate analysis
PD: Faith’s phylogenetic diversity index
LMM: Linear mixed model
WUnifrac: Weighted unifrac
DSI: Dyadic sociality index

## References

[1] Clemente JC, Ursell LK, Parfrey LW, Knight R (2012). The impact of the gut microbiota on human health: An integrative view. Cell.

[2] Cryan JF, O’riordan KJ, Cowan CSM, Sandhu KV, Bastiaanssen TFS, Boehme M, Codagnone MG, Cussotto S, Fulling C, Golubeva AV, Guzzetta KE, Jaggar M, Long-Smith CM, Lyte JM, Martin JA, Molinero-Perez A, Moloney G, Morelli E, Morillas E, O’connor R, Cruz-Pereira JS, Peterson VL, Rea K, Ritz NL, Sherwin E, Spichak S, Teichman EM, van de Wouw M, Ventura-Silva AP, Wallace-Fitzsimons SE, Hyland N, Clarke G, Dinan TG (2019). The microbiota-gut-brain axis. Physiol. Rev..

[3] Parfrey LW, Walters WA, Knight R (2011). Microbial eukaryotes in the human microbiome: Ecology, evolution, and future directions. Front. Microbiol..

[4] Caporaso JG, Lauber CL, Costello EK, Berg-Lyons D, Gonzalez A, Stombaugh J, Knights D, Gajer P, Ravel J, Fierer N, Gordon JI, Knight R (2011). Moving pictures of the human microbiome. Genome Biol..

[5] Björk JR, Dasari M, Grieneisen L, Archie EA (2019). Primate microbiomes over time: Longitudinal answers to standing questions in microbiome research. Am. J. Primatol..

[6] Costello EK, Stagaman K, Dethlefsen L, Bohannan BJM, Relman DA (2012). The application of ecological theory toward an understanding of the human microbiome. Science.

[7] Miller ET, Svanbäck R, Bohannan BJM (2018). Microbiomes as metacommunities: Understanding host-associated microbes through metacommunity ecology. Trends Ecol. Evol..

[8] McKenney EA, Koelle K, Dunn RR, Yoder AD (2018). The ecosystem services of animal microbiomes. Mol. Ecol..

[9] Koskella B, Hall LJ, Metcalf CJE (2017). The microbiome beyond the horizon of ecological and evolutionary theory. Nat. Ecol. Evol..

[10] Sarkar A, Harty S, Johnson KVA, Moeller AH, Archie EA, Schell LD, Carmody RN, Clutton-Brock TH, Dunbar RIM, Burnet PWJ (2020). Microbial transmission in animal social networks and the social microbiome. Nat. Ecol. Evol..

[11] Rothschild D, Weissbrod O, Barkan E, Kurilshikov A, Korem T, Zeevi D, Costea PI, Godneva A, Kalka IN, Bar N, Shilo S, Lador D, Vila AV, Zmora N, Pevsner-Fischer M, Israeli D, Kosower N, Malka G, Wolf BC, Avnit-Sagi T, Lotan-Pompan M, Weinberger A, Halpern Z, Carmi S, Fu J, Wijmenga C, Zhernakova A, Elinav E, Segal E (2018). Environment dominates over host genetics in shaping human gut microbiota. Nature.

[12] Degnan PH, Pusey AE, Lonsdorf EV, Goodall J, Wroblewski EE, Wilson ML, Rudicell RS, Hahn BH, Ochman H (2012). Factors associated with the diversification of the gut microbial communities within chimpanzees from Gombe National Park. Proc. Natl. Acad. Sci..

[13] Bennett G, Malone M, Sauther ML, Cuozzo FP, White B, Nelson KE, Stumpf RM, Knight R, Leigh SR, Amato KR (2016). Host age, social group, and habitat type influence the gut microbiota of wild ring-tailed lemurs (*Lemur catta*). Am. J. Primatol..

[14] Amato KR, Van Belle S, Di Fiore A, Estrada A, Stumpf R, White B, Nelson KE, Knight R, Leigh SR (2017). Patterns in gut microbiota similarity associated with degree of sociality among sex classes of a neotropical primate. Microb. Ecol..

[15] Raulo A, Ruokolainen L, Lane A, Amato K, Knight R, Leigh S, Stumpf R, White B, Nelson KE, Baden AL, Tecot SR (2017). Social behaviour and gut microbiota in red-bellied lemurs (*Eulemur rubriventer*): In search of the role of immunity in the evolution of sociality. J. Anim. Ecol..

[16] Springer A, Fichtel C, Al-Ghalith GA, Koch F, Amato KR, Clayton JB, Knights D, Kappeler PM (2017). Patterns of seasonality and group membership characterize the gut microbiota in a longitudinal study of wild Verreaux’s sifakas (*Propithecus verreauxi*). Ecol. Evol..

[17] Tung J, Barreiro LB, Burns MB, Grenier JC, Lynch J, Grieneisen LE, Altmann J, Alberts SC, Blekhman R, Archie EA (2015). Social networks predict gut microbiome composition in wild baboons. Elife.

[18] Moeller AH, Foerster S, Wilson ML, Pusey AE, Hahn BH, Ochman H (2016). Social behavior shapes the chimpanzee pan-microbiome. Sci. Adv..

[19] Perofsky AC, Lewis RJ, Abondano LA, Di Fiore A, Meyers LA (2017). Hierarchical social networks shape gut microbial composition in wild Verreaux’s sifaka. Proc. R. Soc. B Biol. Sci..

[20] Raulo A, Allen BE, Troitsky T, Husby A, Firth JA, Coulson T, Knowles SCL (2021). Social networks strongly predict the gut microbiota of wild mice. ISME J..

[21] Arrieta MC, Stiemsma LT, Amenyogbe N, Brown E, Finlay B (2014). The intestinal microbiome in early life: Health and disease. Front. Immunol..

[22] Ren T, Grieneisen LE, Alberts SC, Archie EA, Wu M (2016). Development, diet and dynamism: Longitudinal and cross-sectional predictors of gut microbial communities in wild baboons. Environ. Microbiol..

[23] Jagsi R, Jiang J, Momoh AO, Alderman A, Giordano SH, Buchholz TA, Pierce LJ, Kronowitz SJ, Smith BD (2017). Seasonal cycling in the gut microbiome of the Hadza Hunter-Gatherers of Tanzania. Science.

[24] Hicks AL, Lee KJ, Couto-Rodriguez M, Patel J, Sinha R, Guo C, Olson SH, Seimon A, Seimon TA, Ondzie AU, Karesh WB, Reed P, Cameron KN, Lipkin WI, Williams BL (2018). Gut microbiomes of wild great apes fluctuate seasonally in response to diet. Nat. Commun..

[25] Murillo T, Schneider D, Fichtel C, Daniel R (2022). Dietary shifts and social interactions drive temporal fluctuations of the gut microbiome from wild redfronted lemurs. ISME Commun..

[26] Laforest-Lapointe I, Arrieta M-C (2018). Microbial eukaryotes: A missing link in gut microbiome studies. mSystems.

[27] Mann AE, Mazel F, Lemay MA, Morien E, Billy V, Kowalewski M, Di Fiore A, Link A, Goldberg TL, Tecot S, Baden AL, Gomez A, Sauther ML, Cuozzo FP, Rice GAO, Dominy NJ, Stumpf R, Lewis RJ, Swedell L, Amato K, Wegener PL (2020). Biodiversity of protists and nematodes in the wild nonhuman primate gut. ISME J..

[28] Vlčková K, Pafčo B, Petrželková KJ, Modrý D, Todd A, Yeoman CJ, Torralba M, Wilson BA, Stumpf RM, White BA, Nelson KE, Leigh SR, Gomez A (2018). Relationships between gastrointestinal parasite infections and the fecal microbiome in free-ranging western lowland gorillas. Front. Microbiol..

[29] Renelies-Hamilton J, Noguera-Julian M, Parera M, Paredes R, Pacheco L, Dacal E, Saugar JM, Rubio JM, Poulsen M, Köster PC, Carmena D (2019). Exploring interactions between *Blastocystis* sp., *Strongyloides* spp. and the gut microbiomes of wild chimpanzees in Senegal. Infect. Genet. Evol..

[30] Martínez-Mota R, Righini N, Mallott EK, Gillespie TR, Amato KR (2021). The relationship between pinworm (Trypanoxyuris) infection and gut bacteria in wild black howler monkeys (*Alouatta pigra*). Am. J. Primatol..

[31] Pereira ME, Kaufman R, Kappeler PM, Overdoff DJ (1990). Female dominance does not characterize all of the lemuridae. Folia Primatol..

[32] Ostner J, Kappeler PM (1999). Central males instead of multiple pairs in redfronted lemurs, *Eulemur fulvus rufus* (Primates, Lemuridae)?. Anim. Behav..

[33] Kappeler PM, Fichtel C (2012). A 15-year perspective on the social organization and life history of sifaka in Kirindy Forest. Long-Term Field Studies of Primates.

[34] Koch F, Ganzhorn JU, Rothman JM, Chapman CA, Fichtel C (2017). Sex and seasonal differences in diet and nutrient intake in Verreaux’s sifakas (*Propithecus verreauxi*). Am. J. Primatol..

[35] Scholz F, Kappeler PM (2004). Effects of seasonal water scarcity on the ranging behavior of *Eulemur fulvus rufus*. Int. J. Primatol..

[36] Amoroso CR, Kappeler PM, Fichtel C, Nunn CL (2020). Water availability impacts habitat use by red-fronted lemurs (*Eulemur rufifrons*): An experimental and observational study. Int. J. Primatol..

[37] Clough D, Heistermann M, Kappeler PM (2010). Host intrinsic determinants and potential consequences of parasite infection in free-ranging red-fronted lemurs (*Eulemur fulvus rufus*). Am. J. Phys. Anthropol..

[38] Ostner J, Kappeler P, Heistermann M (2008). Androgen and glucocorticoid levels reflect seasonally occurring social challenges in male redfronted lemurs (*Eulemur fulvus rufus*). Behav. Ecol. Sociobiol..

[39] Heistermann M, Palme R, Ganswindt A (2006). Comparison of different enzymeimmunoassays for assessment of adrenocortical activity in primates based on fecal analysis. Am. J. Primatol..

[40] Kappeler PM, Fichtel C (2012). Female reproductive competition in *Eulemur rufifrons*: Eviction and reproductive restraint in a plurally breeding Malagasy primate. Mol. Ecol..

[41] Ostner J, Kappeler PM, Heistermann M (2002). Seasonal variation and social correlates of androgen excretion in male redfronted lemurs (*Eulemur fulvus rufus*). Behav. Ecol. Sociobiol..

[42] Clough D (2010). Gastro-intestinal parasites of red-fronted lemurs in Kirindy Forest, western Madagascar. J. Parasitol..

[43] Gogarten JF, Calvignac-Spencer S, Nunn CL, Ulrich M, Saiepour N, Nielsen HV, Deschner T, Fichtel C, Kappeler PM, Knauf S, Müller-Klein N, Ostner J, Robbins MM, Sangmaneedet S, Schülke O, Surbeck M, Wittig RM, Sliwa A, Strube C, Leendertz FH, Roos C, Noll A (2020). Metabarcoding of eukaryotic parasite communities describes diverse parasite assemblages spanning the primate phylogeny. Mol. Ecol. Resour..

[44] Barton RA (1987). Allogrooming as mutualism in diurnal lemurs. Primates.

[45] Noguera JC, Aira M, Pérez-Losada M, Domínguez J, Velando A (2018). Glucocorticoids modulate gastrointestinal microbiome in a wild bird. R. Soc. Open Sci..

[46] Klindworth A, Pruesse E, Schweer T, Peplies J, Quast C, Horn M, Glöckner FO (2013). Evaluation of general 16S ribosomal RNA gene PCR primers for classical and next-generation sequencing-based diversity studies. Nucleic Acids Res..

[47] Stoeck T, Bass D, Nebel M, Christen R, Jones MDM, Breiner H-W, Richards TA (2010). Multiple marker parallel tag environmental DNA sequencing reveals a highly complex eukaryotic community in marine anoxic water. Mol. Ecol..

[48] Quast C, Pruesse E, Yilmaz P, Gerken J, Schweer T, Yarza P, Peplies J, Glöckner FO (2013). The SILVA ribosomal RNA gene database project: Improved data processing and web-based tools. Nucleic Acids Res..

[49] Yarza P, Yilmaz P, Pruesse E, Glöckner FO, Ludwig W, Schleifer KH, Whitman WB, Euzéby J, Amann R, Rosselló-Móra R (2014). Uniting the classification of cultured and uncultured bacteria and archaea using 16S rRNA gene sequences. Nat. Rev. Microbiol..

[50] Guillou L, Bachar D, Audic S, Bass D, Berney C, Bittner L, Boutte C, Burgaud G, De Vargas C, Decelle J, Del Campo J, Dolan JR, Dunthorn M, Edvardsen B, Holzmann M, Kooistra WHCF, Lara E, Le Bescot N, Logares R, Mahé F, Massana R, Montresor M, Morard R, Not F, Pawlowski J, Probert I, Sauvadet AL, Siano R, Stoeck T, Vaulot D, Zimmermann P, Christen R (2013). The Protist Ribosomal Reference database (PR2): A catalog of unicellular eukaryote small sub-unit rRNA sequences with curated taxonomy. Nucleic Acids Res..

[51] Gao X, Lin H, Revanna K, Dong Q (2017). A Bayesian taxonomic classification method for 16S rRNA gene sequences with improved species-level accuracy. BMC Bioinform..

[52] Reitmeier S, Hitch TCA, Treichel N, Fikas N, Hausmann B, Ramer-Tait AE, Neuhaus K, Berry D, Haller D, Lagkouvardos I, Clavel T (2021). Handling of spurious sequences affects the outcome of high-throughput 16S rRNA gene amplicon profiling. ISME Commun..

[53] Shutt K, Setchell JM, Heistermann M (2012). Non-invasive monitoring of physiological stress in the Western lowland gorilla (*Gorilla gorilla gorilla*): Validation of a fecal glucocorticoid assay and methods for practical application in the field. Gen. Comp. Endocrinol..

[54] Hämäläinen A, Heistermann M, Fenosoa ZSE, Kraus C (2014). Evaluating capture stress in wild gray mouse lemurs via repeated fecal sampling: Method validation and the influence of prior experience and handling protocols on stress responses. Gen. Comp. Endocrinol..

[55] Rudolph K, Fichtel C, Heistermann M, Kappeler PM (2020). Dynamics and determinants of glucocorticoid metabolite concentrations in wild Verreaux’s sifakas. Horm. Behav..

[56] Heitlinger E, Ferreira SCM, Thierer D, Hofer H, East ML (2017). The intestinal eukaryotic and bacterial biome of spotted hyenas: The impact of social status and age on diversity and composition. Front. Cell Infect. Microbiol..

[57] Barr DJ, Levy R, Scheepers C, Tily HJ (2013). Random effects structure for confirmatory hypothesis testing: Keep it maximal. J. Mem. Lang..

[58] Mallick H, Rahnavard A, McIver LJ, Ma S, Zhang Y, Nguyen LH, Tickle TL, Weingart G, Ren B, Schwager EH, Chatterjee S, Thompson KN, Wilkinson JE, Subramanian A, Lu Y, Waldron L, Paulson JN, Franzosa EA, Bravo HC, Huttenhower C (2021). Multivariable association discovery in population-scale meta-omics studies. PLoS Comput. Biol..

[59] De Cáceres M, Legendre P, Moretti M (2010). Improving indicator species analysis by combining groups of sites. Oikos.

[60] Silk J, Cheney D, Seyfarth R (2013). A practical guide to the study of social relationships. Evol. Anthropol..

[61] Ostner J, Nunn CL, Schülke O (2008). Female reproductive synchrony predicts skewed paternity across primates. Behav. Ecol..

[62] Bailey MT, Dowd SE, Galley JD, Hufnagle AR, Allen RG, Lyte M (2011). Exposure to a social stressor alters the structure of the intestinal microbiota: Implications for stressor-induced immunomodulation. Brain Behav. Immun..

[63] Bailey MT, Dowd SE, Parry NMA, Galley JD, Schauer DB, Lyte M (2010). Stressor exposure disrupts commensal microbial populations in the intestines and leads to increased colonization by Citrobacter rodentium. Infect. Immun..

[64] Stothart MR, Bobbie CB, Schulte-Hostedde AI, Boonstra R, Palme R, Mykytczuk NCS, Newman AEM (2016). Stress and the microbiome: Linking glucocorticoids to bacterial community dynamics in wild red squirrels. Biol. Lett..

[65] Vlčková K, Shutt-Phillip K, Heisterman M, Pafčo B, Petrželkov KJ, Todd A, Modrý D, Nelson KE, Wilson BA, Stumpf RM, White BA, Leigh SR, Gomez A (2018). Impact of stress on the gut microbiome of free-ranging western lowland gorillas. Microbiol.

[66] Chu H, Mazmanian SK (2013). Innate immune recognition of the microbiota promotes host-microbial symbiosis. Nat. Immunol..

[67] Zheng D, Liwinski T, Elinav E (2020). Interaction between microbiota and immunity in health and disease. Cell Res..

[68] Ley RE (2016). Prevotella in the gut: Choose carefully. Nat. Rev. Gastroenterol. Hepatol..

[69] Manara S, Asnicar F, Beghini F, Bazzani D, Cumbo F, Zolfo M, Nigro E, Karcher N, Manghi P, Metzger MI, Pasolli E, Segata N (2019). Microbial genomes from non-human primate gut metagenomes expand the primate-associated bacterial tree of life with over 1000 novel species. Genome Biol..

[70] Round JL, Mazmanian SK (2009). The gut microbiota shapes intestinal immune responses during health and disease. Nat. Rev. Immunol..

[71] Maltz RM, Keirsey J, Kim SC, Mackos AR, Gharaibeh RZ, Moore CC, Xu J, Bakthavatchalu V, Somogyi A, Bailey MT (2018). Prolonged restraint stressor exposure in outbred CD-1 mice impacts microbiota, colonic inflammation, and short chain fatty acids. PLoS ONE.

[72] Ostner J, Heistermann M (2003). Endocrine characterization of female reproductive status in wild redfronted lemurs (*Eulemur fulvus rufus*). Gen. Comp. Endocrinol..

[73] Peckre LR, Defolie C, Kappeler PM, Fichtel C (2018). Potential self-medication using millipede secretions in red-fronted lemurs: Combining anointment and ingestion for a joint action against gastrointestinal parasites?. Primates.

[74] Jenkins TP, Rathnayaka Y, Perera PK, Peachey LE, Nolan MJ, Krause L, Rajakaruna RS, Cantacessi C (2017). Infections by human gastrointestinal helminths are associated with changes in faecal microbiota diversity and composition. PLoS ONE.

[75] Rosa BA, Supali T, Gankpala L, Djuardi Y, Sartono E, Zhou Y, Fischer K, Martin J, Tyagi R, Bolay FK, Fischer PU, Yazdanbakhsh M, Mitreva M (2018). Differential human gut microbiome assemblages during soil-transmitted helminth infections in Indonesia and Liberia. Microbiome.

[76] Reynolds LA, Finlay BB, Maizels RM (2015). Cohabitation in the intestine: Interactions among helminth parasites, bacterial microbiota, and host immunity. J. Immunol..

[77] Toro-Londono MA, Bedoya-Urrego K, Garcia-Montoya GM, Galvan-Diaz AL, Alzate JF (2019). Intestinal parasitic infection alters bacterial gut microbiota in children. PeerJ.

[78] Vacca M, Celano G, Calabrese FM, Portincasa P, Gobbetti M, De AM (2020). The controversial role of human gut Lachnospiraceae. Microorganisms.

[79] Wei Z, Xie X, Xue M, Valencak TG, Liu J, Sun H (2021). The effects of non-fiber carbohydrate content and forage type on rumen microbiome of dairy cows. Animals.

[80] Kaakoush NO (2015). Insights into the role of Erysipelotrichaceae in the human host. Front. Cell Infect. Microbiol..

[81] Ricaboni D, Mailhe M, Cadoret F, Vitton V, Fournier PE, Raoult D (2017). ‘*Colidextribacter massiliensis*’ gen. nov., sp. nov., isolated from human right colon. New Microbes New Infect..

[82] Qin P, Zou Y, Dai Y, Luo G, Zhang X, Xiao L (2019). Characterization a novel butyric acid-producing bacterium *Collinsella aerofaciens* subsp. *shenzhenensis* subsp. nov. Microorganisms.

[83] Wei Y, Gao J, Kou Y, Meng L, Zheng X, Liang M, Sun H, Liu Z, Wanga Y (2020). Commensal bacteria impact a protozoan’s integration into the murine gut microbiota in a dietary nutrient-dependent manner. Appl. Environ. Microbiol..

[84] Perofsky AC, Ancel Meyers L, Abondano LA, Di Fiore A, Lewis RJ (2021). Social groups constrain the spatiotemporal dynamics of wild sifaka gut microbiomes. Mol. Ecol..

[85] Pyritz L, Kappeler PM, Fichtel C (2011). Coordination of group movements in wild red-fronted lemurs (*Eulemur rufifrons*): Processes and influence of ecological and reproductive seasonality. Int. J. Primatol..

[86] Amato KR, Yeoman CJ, Kent A, Righini N, Carbonero F, Estrada A, Rex Gaskins H, Stumpf RM, Yildirim S, Torralba M, Gillis M, Wilson BA, Nelson KE, White BA, Leigh SR (2013). Habitat degradation impacts black howler monkey (*Alouatta pigra*) gastrointestinal microbiomes. ISME J..

[87] Hippe H, Hagelstein A, Kramer I, Swiderski J, Stackebrandt E (1999). Phylogenetic analysis of *Formivibrio citricus*, *Propionivibrio dicarboxylicus*, *Anaerobiospirillum thomasii*, *Succinirnonas amylolytica* and *Succinivibrio dextrinosolvens* and proposal of *Succinivibrionaceae* fam. nov. Int. J. Syst. Evol. Microbiol..

[88] Grieneisen LE, Livermore J, Alberts S, Tung J, Archie EA (2017). Group living and male dispersal predict the core gut microbiome in wild baboons. Integr. Comp. Biol..

[89] Amoroso CR, Kappeler PM, Fichtel C, Nunn CL (2019). Fecal contamination, parasite risk, and waterhole use by wild animals in a dry deciduous forest. Behav. Ecol. Sociobiol..

[90] Vandeputte D, Falony G, Vieira-Silva S, Tito RY, Joossens M, Raes J (2016). Stool consistency is strongly associated with gut microbiota richness and composition, enterotypes and bacterial growth rates. Gut.

[91] Falony G, Joossens M, Vieira-Silva S, Wang J, Darzi Y, Faust K, Kurilshikov A, Bonder MJ, Valles-Colomer M, Vandeputte D, Tito RY, Chaffron S, Rymenans L, Verspecht C, De Sutter L, Lima-Mendez G, D’hoe K, Jonckheere K, Homola D, Garcia R, Tigchelaar EF, Eeckhaudt L, Fu J, Henckaerts L, Zhernakova A, Wijmenga C, Raes J (2016). Population-level analysis of gut microbiome variation. Science.

[92] Sonnenburg JL, Bäckhed F (2016). Diet-microbiota interactions as moderators of human metabolism. Nature.

[93] Zmora N, Suez J, Elinav E (2018). You are what you eat: Diet, health and the gut microbiota. Nat. Rev. Gastroenterol. Hepatol..

[94] Ortmann S, Bradley BJ, Stolter C, Ganzhorn JU, Hohmann G, Robbins M, Boesch C (2006). Estimating the quality and composition of wild animal diets—a critical survey of methods. Feeding Ecology in Apes and Other Primates. Ecological, Physical, and Behavioral Aspects.

