# Supplementary material for: Assessing the drivers of gut microbiome composition in wild redfronted lemurs via longitudinal metacommunity analysis
Source: Sci Rep. 2022 Dec 12;12:21462. doi: 10.1038/s41598-022-25733-x (PMC9744850; doi:10.1038/s41598-022-25733-x)
Supplement: Supplementary file 1 — Supplementary Information 1. [file 41598_2022_25733_MOESM1_ESM.docx]

**Title****:** Assessing the drivers of gut microbiome composition in wild redfronted lemurs via longitudinal metacommunity analysis

Tatiana Murillo, Dominik Schneider, Michael Heistermann, Rolf Daniel, and Claudia Fichtel

**SUPPLEMENTARY MATERIAL**

**FIGURES**

**
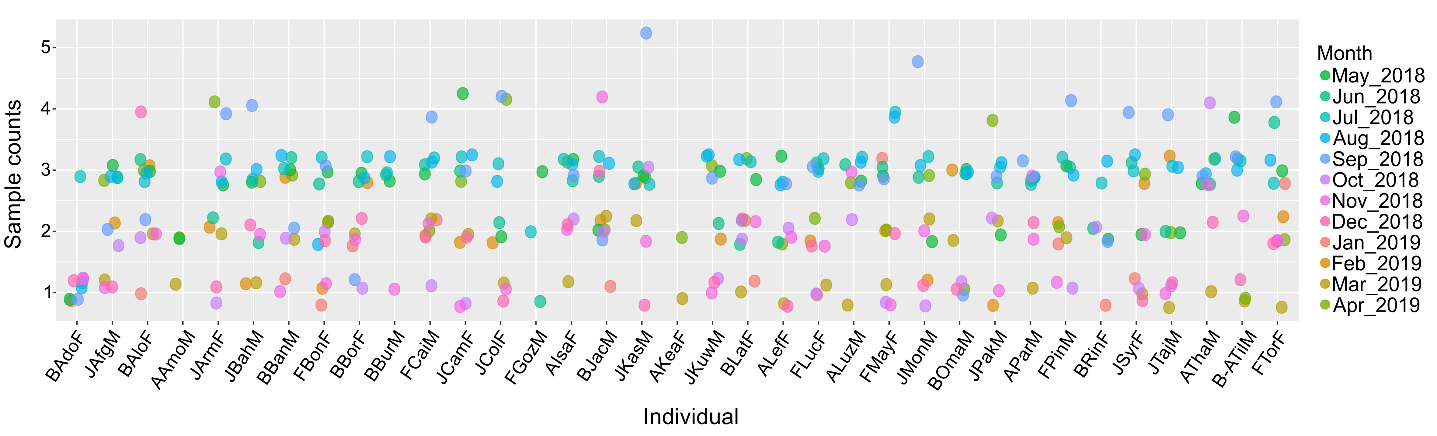
**

**Supplementary Figure S1.** Monthly number of fecal samples collected for each redfronted lemur individual belonging to the four studied groups from May 2018 until April 2019.

**
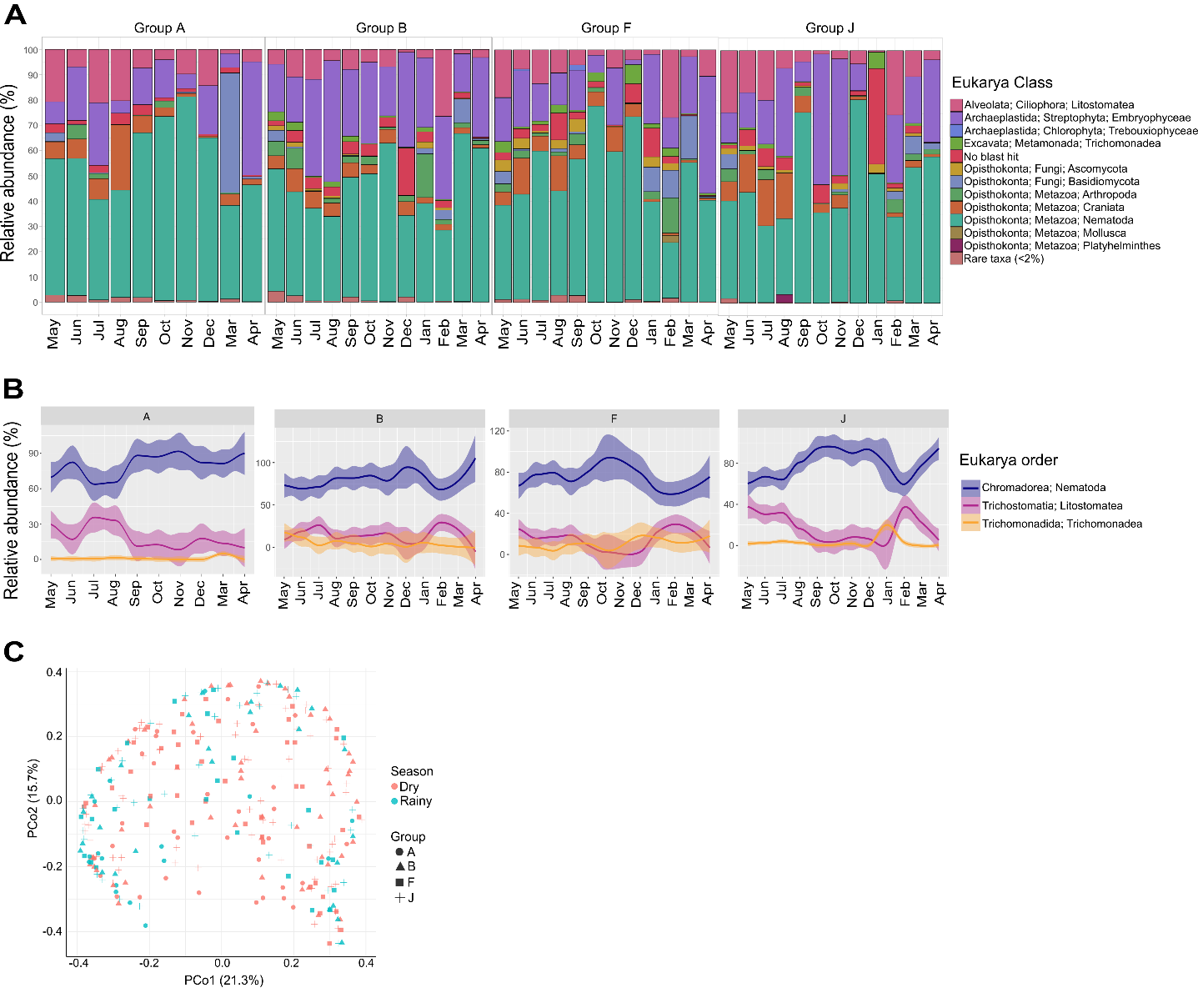
**

**Supplementary Figure S2.** Eukaryotic organisms detected in redfronted lemur fecal samples by using 18S rRNA gene sequencing. **A.** Monthly relative abundances of the eukaryotic organisms detected per lemur group. **B.** Monthly fluctuations in the relative abundances of the previously reported parasites of redfronted lemurs: *Chromadorea*, *Trichostomatia* and *Trichomonadida*. **C.** PCoA based on Jaccard distance matrix of eukaryotic endoparasites detected in redfronted lemurs with data points color coded to season. **D.** PCoA based on Jaccard distance matrix of eukaryotic endoparasites detected in redfronted lemurs with data points color coded to group.

**
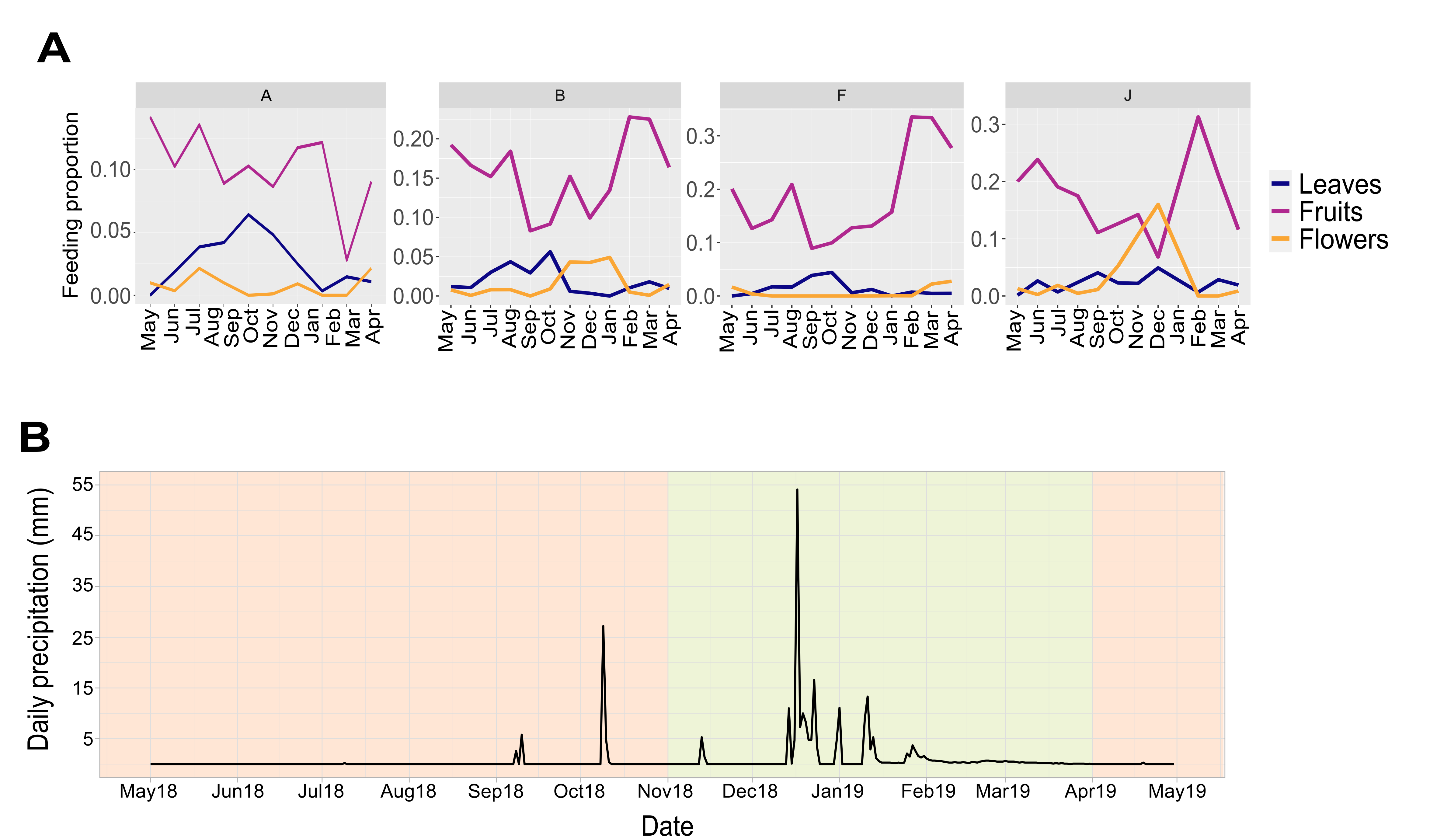
**

**Supplementary Figure S3.** Food items consumed by redfronted lemurs and daily precipitation in Kirindy Forest recorded during the study period from May 2018 until April 2019. **A.** Monthly proportion of time spent feeding on fruits, leaves and flowers 30 days prior to sampling for each group. **B.** Daily precipitation measured in mm during the study period. Color coded panels indicate the dry (brown) and rainy (green) season.


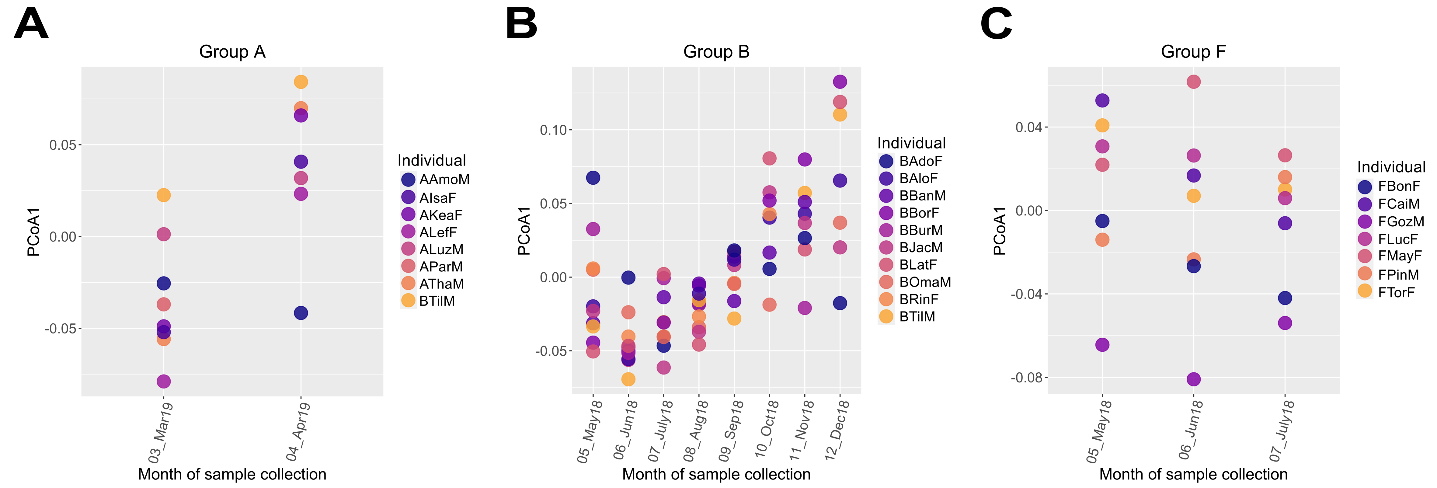


**Supplementary Figure S4.** Comparison of monthly fluctuations in bacterial community composition between resident and migrating individuals. Monthly samples for each individual were merged and a PCoA was calculated from Weighted Unifrac distances. The first coordinate of the PCoA was plotted against months when all individuals were present. Each individual from each group is depicted with a different color. **A.** Group A (migrating individuals: AAmoM & ATilM). **B.** Group B (migrating individuals: BAdoF, BBurM, BRinF & ATilM). **C.** Group F (migrating individuals: FGozM).

**TABLES**

**Supplementary Table S1.** Fecal sample list for 16S rRNA analysis with metadata. Available at figshare: https://doi.org/10.6084/m9.figshare.16974673.v1.

**Supplementary Table S2.** Fecal sample list for 18S rRNA analysis. Available at figshare: https://doi.org/10.6084/m9.figshare.16974742.v2.

**Supplementary Table S3.** Primers and PCR protocols for the studied taxonomical marker genes.

| **Taxonomical marker gene** | **Bacteria 16S rRNA** | **Eukaryota 18S rRNA** |
| --- | --- | --- |
| **Name - Primer Forward** | S-D-Bact-0341-b-S-17 | Reuk454FWD1 |
| **Sequence - Primer Forward** | 5´-CCTACGGGNGGCWGCAG-3´ | 5´-CCAGCASCYGCGGTAATTCC-3´ |
| **Miseq Adapter - Forward** | 5´-TCGTCGGCAGCGTCAGATGTGTATAAGAGACAG-3´ | 5´-TCGTCGGCAGCGTCAGATGTGTATAAGAGACAG-3´ |
| **Name - Primer Reverse** | S-D-Bact-0785-a-A-21 | TAReukREV3 |
| **Sequence - Primer reverse** | 5´-GACTACHVGGGTATCTAATCC-3´ | 5´-ACTTTCGTTCTTGATYRA-3´ |
| **Miseq Adapter - Reverse** | 5´-GTCTCGTGGGCTCGGAGATGTGTATAAGAGACAG-3´ | 5´-GTCTCGTGGGCTCGGAGATGTGTATAAGAGACAG-3´ |
| **PCR protocol** | Final volume of 50 µl containing 10 µl of 5x GC Buffer (Thermo Scientific, Waltham, MA, USA), 5% DMSO, 0.2 mM of forward and reverse primer, 200 µM dNTPs, 0.2 mM MgCl2, 1 U Phusion High-Fidelity DNA polymerase (Thermo Scientific, Waltham, MA, USA) and 20–25 ng template DNA | Final volume of 50 µl containing 10 µl of 5x GC Buffer (Thermo Scientific, Waltham, MA, USA), 5% DMSO, 0.2 mM of forward and reverse primer, 200 µM dNTPs, 0.2 mM MgCl2, 1 U Phusion High-Fidelity DNA polymerase (Thermo Scientific, Waltham, MA, USA) and 50 ng template DNA |
| **Thermocycling program** | Denaturation 1 min at 98 °C, 25 cycles at 98 °C for 45 s, 45 s at 55 °C, and 30 s at 72 °C, and final extension at 72 °C for 5 min. | Denaturation 1 min at 98 °C, 25 cycles at 98 °C for 45 s, 45 s at 47 °C, and 30 s at 72 °C, and final extension at 72 °C for 5 min. |
| **References** | Klindworth et al., 2013 | Stoeck et al., 2010 |
| **Positive control** | *Escherichia coli* | *Aspergillus nidulans* |

**Supplementary Table S4.** Sequencing statistics for 16S rRNA and 18S rRNA.

| **Taxonomical marker gene** | **Bacteria 16S rRNA** | **Eukaryota 18S rRNA** |
| --- | --- | --- |
| **Number of samples** | 799 | 380 |
| **Reads after quality filtering** | 35 801 327 | 21 949 694 |
| **Number of ASVs** | 7 213 | 6 245 |
| **Mean amplicon length (bp)** | 416.44 | 380.81 |
| **Reads after 0.25% filtering** | 32 343 875 | 21 430 144 |
| **Number of ASVs after 0.25% filtering** | 1 028 | 783 |
| **Unclassified reads after 0.25% filtering** | 0.03% ± 0.4 | 3.22% ± 2.10 |
| **Reads for rarefaction** | 8 236 | 6 222 |

**Supplementary Table S5.** ASVs obtained for the 16S rRNA amplicon sequencing. Available at figshare: https://doi.org/10.6084/m9.figshare.16974790.v1.

**Supplementary Table S6.** ASVs obtained for the 18S rRNA amplicon sequencing. Available at figshare: https://doi.org/10.6084/m9.figshare.16974802.v1.

**Supplementary Table S7.** Results PERMANOVA test of Jaccard distance matrix for parasites.

|  | Df | SumsOfSqs | MeanSqs | F.Model | R2 | Pr(>F) | BH |
| --- | --- | --- | --- | --- | --- | --- | --- |
| group | 3 | 3.905 | 1.302 | 4.025 | 0.038 | 0.001 | 0.002 |
| sex | 1 | 0.289 | 0.289 | 0.893 | 0.003 | 0.621 | 0.621 |
| age_months | 1 | 0.663 | 0.663 | 2.051 | 0.007 | 0.004 | 0.005 |
| season | 1 | 2.757 | 2.757 | 8.525 | 0.027 | 0.001 | 0.002 |
| Residuals | 291 | 94.104 | 0.323 | NA | 0.925 | NA | NA |
| Total | 297 | 101.718 | NA | NA | 1 | NA | NA |

**Supplementary Table S8.** Estimates LMM alpha diversity of the full dataset.

| Model comparison | AIC | logLik | Chisq | Df | p value |
| --- | --- | --- | --- | --- | --- |
| Null | 5181.749 | -2548.87 | NA | NA | NA |
| Full | 5183.354 | -2542.68 | 12.3948 | 7 | 0.0883 |
|  |  |  |  |  |  |

|  | Estimate | Std. Error | df | t value | Pr(>\|t\|) |
| --- | --- | --- | --- | --- | --- |
| (Intercept) | 26.124 | 1.641 | 29.235 | 15.915 | 0.000 |
| sexmale | 1.153 | 0.924 | 94.322 | 1.248 | 0.215 |
| groupB | -0.622 | 1.859 | 31.946 | -0.335 | 0.740 |
| groupF | -5.333 | 1.957 | 46.097 | -2.725 | 0.009 |
| groupJ | -2.116 | 1.808 | 32.113 | -1.170 | 0.251 |
| z.log.age_months* | -0.055 | 0.466 | 178.530 | -0.118 | 0.906 |
| z.soc.int* | 0.869 | 0.486 | 11.289 | 1.786 | 0.101 |
| z.ffr.prop* | -0.614 | 0.523 | 29.117 | -1.173 | 0.250 |
| z.fle.prop* | 2.243 | 0.412 | 59.801 | 5.447 | 0.000 |
| z.rain* | -0.985 | 0.546 | 6.504 | -1.805 | 0.117 |
| z.ffl.prop* | -0.169 | 0.637 | 10.635 | -0.265 | 0.796 |
| z.richness.para* | -1.023 | 0.649 | 23.098 | -1.577 | 0.128 |

*The mean and the standard deviation of the original z transformed variables were: log.age_months: 3.929 ± 0.902, soc.int: 0.004 ± 0.009, rain: 0.266 ± 0.557, fle.prop: 0.022 ± 0.033, ffr.prop: 0.159 ± 0.116 , ffl.prop: 0.013 ± 0.039 and richness.para: 48.241 ± 27.076.

**Supplementary Table S9.** Estimates LMM alpha diversity of the dataset including fGCM values.

| Model comparison | AIC | logLik | Chisq | Df | p value |
| --- | --- | --- | --- | --- | --- |
| Null | 4201.814 | -2049.907 | NA | NA | NA |
| Full | 4201.562 | -2041.781 | 16.252 | 8 | 0.039 |
|  |  |  |  |  |  |
|  | Estimate | Std. Error | df | t value | Pr(>\|t\|) |
| (Intercept) | 26.786 | 1.506 | 31.021 | 17.782 | 0.000 |
| sexmale | 0.801 | 0.997 | 197.154 | 0.804 | 0.423 |
| groupB | 0.074 | 1.728 | 31.751 | 0.043 | 0.966 |
| groupF | -5.210 | 1.907 | 53.987 | -2.733 | 0.008 |
| groupJ | -1.777 | 1.711 | 34.705 | -1.038 | 0.306 |
| z.log.age_months* | -0.001 | 0.510 | 204.977 | -0.002 | 0.999 |
| z.soc.int* | 0.613 | 0.618 | 8.713 | 0.992 | 0.348 |
| z.rain* | 0.751 | 0.720 | 19.765 | 1.043 | 0.310 |
| z.fle.prop* | 2.205 | 0.556 | 28.125 | 3.969 | 0.000 |
| z.ffr.prop* | -0.440 | 0.580 | 27.561 | -0.757 | 0.455 |
| z.ffl.prop* | -0.700 | 0.560 | 6.979 | -1.250 | 0.252 |
| z.log.fgc* | 1.216 | 0.532 | 45.259 | 2.285 | 0.027 |
| z.richness.para* | -0.871 | 0.653 | 28.834 | -1.334 | 0.193 |

*The mean and the standard deviation of the original z transformed variables were: log.age_months: 3.911 ± 0.915, soc.int: 0.004 ± 0.008, rain: 0.238 ± 0.470, fle.prop: 0.023 ± 0.033, ffr.prop: 0.153 ± 0.109 , ffl.prop: 0.014 ± 0.042, log.fgc: 5.411 ± 0.868 and richness.para: 47.674 ± 27.061.

**Supplementary Table S10.** Results PERMANOVA test of Wunifrac matrix for dataset with feeding and social interaction data.

|  | Df | SumsOfSqs | MeanSqs | F.Model | R2 | Pr(>F) | BH |
| --- | --- | --- | --- | --- | --- | --- | --- |
| group | 3 | 0.305 | 0.102 | 9.641 | 0.035 | 0.000 | 0.000 |
| soc_int | 1 | 0.037 | 0.037 | 3.553 | 0.004 | 0.012 | 0.014 |
| sex | 1 | 0.047 | 0.047 | 4.442 | 0.005 | 0.000 | 0.000 |
| age_months | 1 | 0.017 | 0.017 | 1.576 | 0.002 | 0.074 | 0.074 |
| rain | 1 | 0.186 | 0.186 | 17.695 | 0.021 | 0.000 | 0.000 |
| fle_prop | 1 | 0.041 | 0.041 | 3.866 | 0.005 | 0.002 | 0.003 |
| ffl_prop | 1 | 0.073 | 0.073 | 6.934 | 0.008 | 0.000 | 0.000 |
| ffr_prop | 1 | 0.076 | 0.076 | 7.192 | 0.009 | 0.000 | 0.000 |
| Residuals | 762 | 8.025 | 0.011 | NA | 0.911 | NA | NA |
| Total | 772 | 8.806 | NA | NA | 1 | NA | NA |

**Supplementary Table S11.** Results PERMANOVA test of Wunifrac matrix for dataset including parasite richness.

|  | Df | SumsOfSqs | MeanSqs | F.Model | R2 | Pr(>F) | BH |
| --- | --- | --- | --- | --- | --- | --- | --- |
| group | 3 | 0.295 | 0.098 | 10.161 | 0.041 | 0.000 | 0.000 |
| soc_int | 1 | 0.040 | 0.040 | 4.152 | 0.006 | 0.004 | 0.004 |
| sex | 1 | 0.036 | 0.036 | 3.754 | 0.005 | 0.000 | 0.000 |
| age_months | 1 | 0.014 | 0.014 | 1.438 | 0.002 | 0.004 | 0.004 |
| richness_para | 1 | 0.046 | 0.046 | 4.715 | 0.006 | 0.000 | 0.000 |
| rain | 1 | 0.177 | 0.177 | 18.306 | 0.024 | 0.000 | 0.000 |
| fle_prop | 1 | 0.037 | 0.037 | 3.790 | 0.005 | 0.002 | 0.002 |
| ffl_prop | 1 | 0.040 | 0.040 | 4.123 | 0.006 | 0.000 | 0.000 |
| ffr_prop | 1 | 0.066 | 0.066 | 6.824 | 0.009 | 0.000 | 0.000 |
| Residuals | 670 | 6.483 | 0.010 | NA | 0.896 | NA | NA |
| Total | 681 | 7.234 | NA | NA | 1 | NA | NA |

**Supplementary Table S12.** Results PERMANOVA test of Wunifrac matrix for dataset including fGCM values.

|  | Df | SumsOfSqs | MeanSqs | F.Model | R2 | Pr(>F) | BH |
| --- | --- | --- | --- | --- | --- | --- | --- |
| group | 3 | 0.310 | 0.103 | 10.853 | 0.052 | 0.000 | 0.000 |
| soc_int | 1 | 0.031 | 0.031 | 3.238 | 0.005 | 0.021 | 0.021 |
| sex | 1 | 0.040 | 0.040 | 4.199 | 0.007 | 0.000 | 0.000 |
| age_months | 1 | 0.018 | 0.018 | 1.876 | 0.003 | 0.001 | 0.001 |
| n11oxo_CM_wet_feces | 1 | 0.167 | 0.167 | 17.513 | 0.028 | 0.000 | 0.000 |
| rain | 1 | 0.134 | 0.134 | 14.044 | 0.022 | 0.000 | 0.000 |
| richness_para | 1 | 0.033 | 0.033 | 3.449 | 0.006 | 0.000 | 0.000 |
| fle_prop | 1 | 0.024 | 0.024 | 2.494 | 0.004 | 0.012 | 0.013 |
| ffl_prop | 1 | 0.036 | 0.036 | 3.762 | 0.006 | 0.001 | 0.001 |
| ffr_prop | 1 | 0.072 | 0.072 | 7.558 | 0.012 | 0.000 | 0.000 |
| Residuals | 534 | 5.078 | 0.010 | NA | 0.855 | NA | NA |
| Total | 546 | 5.941 | NA | NA | 1 | NA | NA |

**Supplementary Table S13.** Results from MaAsLin2 analysis detecting associations between bacterial genera and group membership, social interactions, parasite richness, sex, age, diet, and precipitation. Available at figshare: https://doi.org/10.6084/m9.figshare.16974826.v1.

**Supplementary Table S14.** Results from MaAsLin2 analysis detecting associations between bacterial genera and group membership, social interactions, parasite richness, sex, age, fGCM levels, diet, and precipitation. Available at figshare: https://doi.org/10.6084/m9.figshare.16974832.v2.

**Supplementary Table S15.** Results indicative species analysis for the individuals of group A. Available at figshare: <https://doi.org/10.6084/m9.figshare.16974844.v2>.

**Supplementary Table S16.** DSI for the individuals of group A.

|  | **Amorgos** | **Isabella** | **Kea** | **Lefkada** | **Luzon** | **Paros** | **Thassos** | **Tilos** |
| --- | --- | --- | --- | --- | --- | --- | --- | --- |
| **Amorgos** | NA | 0.2010 | 0.0000 | 0.0176 | 0.4132 | 0.0000 | 0.0470 | 0.0000 |
| **Isabella** | 0.2010 | NA | 1.0000 | 0.4242 | 0.9361 | 0.4426 | 0.5532 | 0.0979 |
| **Kea** | 0.0000 | 1.0000 | NA | 0.1232 | 0.1194 | 0.0000 | 0.1110 | 0.3244 |
| **Lefkada** | 0.0176 | 0.4242 | 0.1232 | NA | 0.4757 | 0.2087 | 0.6823 | 0.0079 |
| **Luzon** | 0.4132 | 0.9361 | 0.1194 | 0.4757 | NA | 0.1571 | 0.3201 | 0.0014 |
| **Paros** | 0.0000 | 0.4426 | 0.0000 | 0.2087 | 0.1571 | NA | 0.0775 | 0.0000 |
| **Thassos** | 0.0470 | 0.5532 | 0.1110 | 0.6823 | 0.3201 | 0.0775 | NA | 0.1547 |
| **Tilos** | 0.0000 | 0.0979 | 0.3244 | 0.0079 | 0.0014 | 0.0000 | 0.1547 | NA |

**Supplementary Table S17.** Results indicative species analysis for the individuals of group B. Available at figshare: https://doi.org/10.6084/m9.figshare.16974856.v2.

**Supplementary Table S18.** DSI for the individuals of group B.

|  | **Adonara** | **Aloha** | **Bangladesh** | **Bora** | **Buru** | **Jaco** | **Latalata** | **Oman** | **Rinca** | **Tilos** |
| --- | --- | --- | --- | --- | --- | --- | --- | --- | --- | --- |
| **Adonara** | NA | 0.4048 | 0.1202 | 0.1436 | 0.0564 | 0.0402 | 0.2427 | 0.2386 | 1.0000 | 0.0353 |
| **Aloha** | 0.4048 | NA | 0.1470 | 0.1322 | 0.0054 | 0.3834 | 0.4534 | 0.3077 | 0.0489 | 0.0579 |
| **Bangladesh** | 0.1202 | 0.1470 | NA | 0.2953 | 0.0169 | 0.1352 | 0.4607 | 0.5225 | 0.2010 | 0.0417 |
| **Bora** | 0.1436 | 0.1322 | 0.2953 | NA | 0.0626 | 0.2230 | 0.6261 | 0.5161 | 0.2155 | 0.1856 |
| **Buru** | 0.0564 | 0.0054 | 0.0169 | 0.0626 | NA | 0.0331 | 0.2438 | 0.1426 | 0.1304 | 0.2499 |
| **Jaco** | 0.0402 | 0.3834 | 0.1352 | 0.2230 | 0.0331 | NA | 0.5213 | 0.2382 | 0.1793 | 0.2037 |
| **Latalata** | 0.2427 | 0.4534 | 0.4607 | 0.6261 | 0.2438 | 0.5213 | NA | 0.9415 | 0.4400 | 0.1001 |
| **Oman** | 0.2386 | 0.3077 | 0.5225 | 0.5161 | 0.1426 | 0.2382 | 0.9415 | NA | 0.3416 | 0.3332 |
| **Rinca** | 1.0000 | 0.0489 | 0.2010 | 0.2155 | 0.1304 | 0.1793 | 0.4400 | 0.3416 | NA | 0.0810 |
| **Tilos** | 0.0353 | 0.0579 | 0.0417 | 0.1856 | 0.2499 | 0.2037 | 0.1001 | 0.3332 | 0.0810 | NA |

**Supplementary Table S19.** Results indicative species analysis for the individuals of group F. Available at figshare: https://doi.org/10.6084/m9.figshare.16974886.v2.

**Supplementary Table S20.** DSI for the individuals of group F.

|  | **Bonacca** | **Caicos** | **Gozo** | **Lucia** | **Mayaguana** | **Pinos** | **Tortuga** |
| --- | --- | --- | --- | --- | --- | --- | --- |
| **Bonacca** | NA | 0.1999 | 0.1165 | 0.8183 | 0.3858 | 0.4174 | 1.0000 |
| **Caicos** | 0.1999 | NA | 0.1373 | 0.6796 | 0.2393 | 0.8149 | 0.2270 |
| **Gozo** | 0.1165 | 0.1373 | NA | 0.1745 | 0.1257 | 0.1583 | 0.2246 |
| **Lucia** | 0.8183 | 0.6796 | 0.1745 | NA | 0.3382 | 0.3439 | 0.6008 |
| **Mayaguana** | 0.3858 | 0.2393 | 0.1257 | 0.3382 | NA | 0.2460 | 0.3943 |
| **Pinos** | 0.4174 | 0.8149 | 0.1583 | 0.3439 | 0.2460 | NA | 0.4919 |
| **Tortuga** | 1.0000 | 0.2270 | 0.2246 | 0.6008 | 0.3943 | 0.4919 | NA |

**Supplementary Table S21.** Results indicative species analysis for the individuals of group J. Available at figshare: https://doi.org/10.6084/m9.figshare.16974889.v2.

**Supplementary Table S22.** DSI for the individuals of group J.

|  | **Afganistan** | **Armenia** | **Bahrain** | **Cambodia** | **Colanta** | **Kasachstan** | **Kuwait** | **Mongolei** | **Pakistan** | **Syria** | **Taji** |
| --- | --- | --- | --- | --- | --- | --- | --- | --- | --- | --- | --- |
| **Afganistan** | NA | 0.0925 | 0.0735 | 0.1543 | 0.0868 | 0.1617 | 0.2804 | 0.0809 | 0.1722 | 0.7911 | 0.2681 |
| **Armenia** | 0.0925 | NA | 0.2752 | 0.0696 | 1.0000 | 0.1305 | 0.0917 | 0.0832 | 0.2946 | 0.3067 | 0.0975 |
| **Bahrain** | 0.0735 | 0.2752 | NA | 0.4200 | 0.2820 | 0.5363 | 0.7211 | 0.5393 | 0.3814 | 0.1897 | 0.2137 |
| **Cambodia** | 0.1543 | 0.0696 | 0.4200 | NA | 0.3508 | 0.3440 | 0.3376 | 0.2530 | 0.1743 | 0.0977 | 0.0520 |
| **Colanta** | 0.0868 | 1.0000 | 0.2820 | 0.3508 | NA | 0.1211 | 0.6606 | 0.4099 | 0.4449 | 0.3987 | 0.3518 |
| **Kasachstan** | 0.1617 | 0.1305 | 0.5363 | 0.3440 | 0.1211 | NA | 0.2490 | 0.3939 | 0.4639 | 0.0077 | 0.2036 |
| **Kuwait** | 0.2804 | 0.0917 | 0.7211 | 0.3376 | 0.6606 | 0.2490 | NA | 0.2811 | 0.6428 | 0.1841 | 0.2314 |
| **Mongolei** | 0.0809 | 0.0832 | 0.5393 | 0.2530 | 0.4099 | 0.3939 | 0.2811 | NA | 0.2257 | 0.1461 | 0.1788 |
| **Pakistan** | 0.1722 | 0.2946 | 0.3814 | 0.1743 | 0.4449 | 0.4639 | 0.6428 | 0.2257 | NA | 0.1220 | 0.0870 |
| **Syria** | 0.7911 | 0.3067 | 0.1897 | 0.0977 | 0.3987 | 0.0077 | 0.1841 | 0.1461 | 0.1220 | NA | 0.2662 |
| **Taji** | 0.2681 | 0.0975 | 0.2137 | 0.0520 | 0.3518 | 0.2036 | 0.2314 | 0.1788 | 0.0870 | 0.2662 | NA |
